# Developing Picornaviruses for Cancer Therapy

**DOI:** 10.3390/cancers11050685

**Published:** 2019-05-16

**Authors:** Cormac McCarthy, Nadishka Jayawardena, Laura N. Burga, Mihnea Bostina

**Affiliations:** 1 Department of Microbiology and Immunology, University of Otago, Dunedin 9016, New Zealand; mccco791@student.otago.ac.nz (C.M.); jaygi915@student.otago.ac.nz (N.J.); 2 Otago Micro and Nano Imaging, University of Otago, Dunedin 9016, New Zealand

**Keywords:** picornavirus, oncolytic virotherapy, poliovirus, coxsackievirus, senecavirus

## Abstract

Oncolytic viruses (OVs) form a group of novel anticancer therapeutic agents which selectively infect and lyse cancer cells. Members of several viral families, including *Picornaviridae*, have been shown to have anticancer activity. Picornaviruses are small icosahedral non-enveloped, positive-sense, single-stranded RNA viruses infecting a wide range of hosts. They possess several advantages for development for cancer therapy: Their genomes do not integrate into host chromosomes, do not encode oncogenes, and are easily manipulated as cDNA. This review focuses on the picornaviruses investigated for anticancer potential and the mechanisms that underpin this specificity.

## 1. Introduction

**Figure 1:**
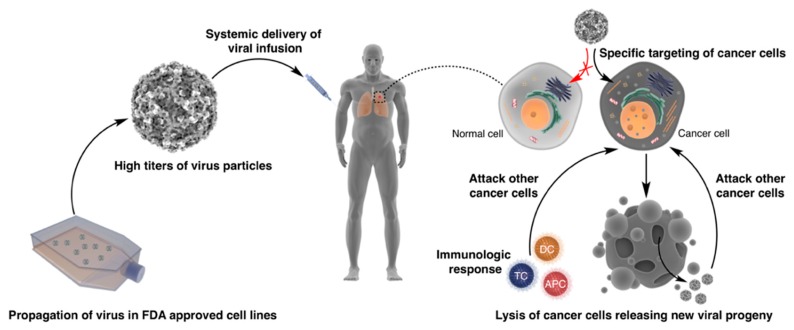
Oncolytic picornaviruses as a cancer treatment. The use of oncolytic picornaviruses is highlighted by their relative ease of production in high titres in food and drug administration (FDA)-approved cell lines. Resulting mature virions can then be systemically administered into a patient with a tumour. Most picornaviruses display a high tropism to a variety of tumours but not to normal tissues, resulting in intratumoural replication and subsequent cellular death. The new viral progeny released from lysed cancer cells can then infect neighbouring cancer cells. The mere presence of virus particles and virus-induced tumour cell death modulates the immune system to activate antigen-presenting cells (APC), denditric cells (DC), and T cells (TC), which collectively provide a long-lasting antitumour activity.

Cancer remains one of the leading causes of death worldwide. Globally, there were around 18 million new cases of cancer in 2018, with up to 9.5 million deaths [1]. Contemporary treatment for cancer usually involves some combination of surgical resection, radiation therapy and/or chemotherapy. While these conventional therapies have improved the prognosis for many cancers, the side-effects are often severe. In addition, some tumours are inoperable or resistant to radio- and chemotherapy, emphasising the need to develop new therapeutic strategies. Oncolytic virotherapy has emerged as a promising way of treating cancers. Broadly speaking, oncolytic viruses are naturally occurring or engineered viruses that selectively infect and lyse cancer cells. Their replication in cancer cells produces progeny viruses which in turn repeat the infection process in neighbouring cells. Furthermore, viral infection can stimulate cytotoxic immune responses against antigen from destroyed cells (Figure 1). The efficacy of oncolytic viruses has been shown in many cases to be increased when used in combination with other therapeutic agents. For example, in mouse models of head and neck cancers, *Herpes simplex* virus-1 has shown increased efficacy when combined with cisplatin [2], and coxsackievirus A21 has greater anti-tumour activity when combined with anti-programmed cell death protein 1 (PD-1) antibodies against metastatic melanoma, bladder cancer, and non-small-cell lung cancer [3]. Oncolytic virotherapy’s roots extend back to early reports of patients with leukaemia and Hodgkin’s disease, who survived concomitant viral infections and showed evidence of clinical remission [4,5]. These observations led to a number of trials from the mid-to-late 20th century, such as West Nile Virus Egypt 101 against various cancers [6], Adenovirus versus cervical carcinomas [7] and Mumps virus against various terminal cancers [8]. Each of these trials showed varying degrees of protective effect. For instance, the trial concerning Adenovirus showed that 26 of 40 inoculations resulted in tumour necrosis [7], and the Mumps virus trial resulted in complete regression of 37 out of 90 trial subjects, as well as 42 instances of growth suppression [8]. In 2015, an important milestone in the endeavour to make oncolytic virotherapy a viable therapy was reached. 

This was the approval of the first oncolytic virus, modified *Herpes simplex* virus-1, also known as Talimogene Laherparepvec (T-VEC), by the US Food and Drug Administration (FDA) for the treatment of malignant melanomas [9]. In addition, Oncorine, derived from adenovirus, was approved in China in 2005 for the treatment of head and neck cancers [10,11]. While cancer cells are generally observed to be more susceptible to viral infection than healthy cells, the clinical use of oncolytic viruses is still relatively modest [12]. This is due to several limiting conditions: Oncolytic viruses have to be nonpathogenic and genetically stable, while still maintaining infectivity to nonhomogenous cancerous cells [13]. They also have to be able to overcome the unfavourable conditions of the tumour microenvironment, including dense connective tissue, poor lymphatic flow and increased interstitial pressure [14]. 

Despite the inherent challenges presented in producing safe and effective oncolytic viruses, there are a number of naturally occurring and engineered viruses that have entered clinical trials. Included in their ranks are Adenovirus engineered to express granulocyte macrophage colony-stimulating factor (GM-CSF) (ONCOS-102^®^), Vaccinia virus engineered to express GM-CSF with thymidine kinase deleted (PexaVec^®^), Measles virus engineered to express thyroidal sodium iodide importer (MV-NIS^®^) and naturally occurring Reovirus (Reolysin^®^). These viruses vary greatly with respect to several characteristics, including the species, genera, etc. to which they belong, their progress through clinical trials, cancer types they are used to treat and degree to which they have been engineered. 

There are a number of ways to increase the effectiveness of oncolytic viruses. Often, they are used in combination with chemotherapeutics and/or radiation/surgical techniques. For instance, Reolysin^®^ in clinical trials has been combined with carboplatin and paclitaxel for the treatment of malignant melanomas [15], and PexaVec^®^ in clinical trials has been combined with sorafenib to combat hepatocellular carcinoma [16]. Oncolytic viruses can also be engineered to express tumour-specific peptides, with the purpose of magnifying the immune response against the tumour. An example of this is vesicular stomatitis virus (VSV) expressing ovalbumin (OVA), being used to greater effect than the parental VSV to treat OVA-expressing B16 melanoma tumours [17]. It is worth mentioning that the utility of engineered VSV extends beyond OVA expression to include expression of immunostimulatory proteins such as IL-4, alternative receptors like Sindbis virus glycoproteins and suicide genes like thymidine kinase [18]. Table 1 provides a brief summary of those oncolytic viruses which have been shown in clinical trials to be especially promising. 

**Figure 2:**
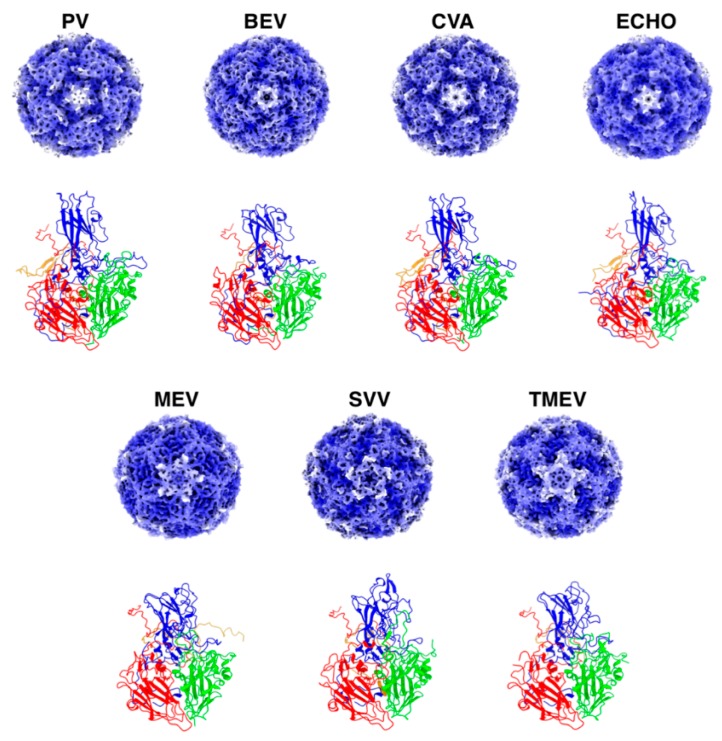
Structures of picornaviruses used in oncovirotherapy. Molecular structures of Type I Poliovirus (PV, PDB ID: 1HXS), Bovine Enterovirus 2 (BEV, PDB ID: 1BEV), Coxsackievirus A21 (CVA, PDB ID: 1Z7S), Echovirus 7 (ECHO, PDB ID: 2X5I), Mengovirus (MEV, PDB ID: 2MEV), Seneca Valley virus (SVV, PDB ID: 3CJI), Theiler’s Murine Encephalomyelitis virus (TMEV, PDB ID: 1TME) and their corresponding protomers. Peaks, intermediate elevations and valleys on depth-cued capsid surfaces are coloured in white, purple and blue, respectively. In capsid protomers, VP1, VP2, VP3 and VP4 are coloured in blue, green, red and yellow, respectively. Figures were generated on ChimeraX [20]. Comparison between the structures can also be seen in the movie: morphs between depth-cued atomic structures of oncolytic picornaviruses (Supplementary Movie).

Derived from the Latin “pico”, meaning small, Picornaviridae members have non-enveloped icosahedral capsids about 30 nm in diameter protecting a small, positive-sense, single-stranded RNA genome (Figure 2). Picornaviruses are among the most researched of viral families, with notable members such as Poliovirus, Foot-and-Mouth Disease virus, Hepatitis A virus and, more recently, Seneca Valley virus. Their genome follows a conserved L-4-3-4 format, where the single polyprotein is cleaved by virally encoded proteases into the Leader protein (present only in some species), four structural and seven nonstructural proteins [21]. Picornaviral genomes start with a 5′ untranslated region (UTR), which associates with the viral genome associated protein (VPg), and includes the internal ribosome entry site (IRES) [22]. Whereas human cellular RNA translation relies on the 7-methyl guanosine cap interacting with the eukaryotic initiation factor (eIF) protein, this function in the cap-independent translation of picornaviral genomes is performed by the IRES [23]. Adjacent to the IRES, the Leader protein is a protease that sits at the 5’ extreme of the translated picornaviral polyprotein, though it is not present in all members of the *Picornaviridae* family. This is followed by the P1 region of the polyprotein, encoding in order the capsid proteins VP4, VP2, VP3 and VP1 respectively [24,25]. While VP1 and VP3 are separated from P1 proteolytically, VP4 and VP2 are usually processed from the precursor VP0 following genome packaging inside the viral capsid (Figure 2).

**Figure 3:**
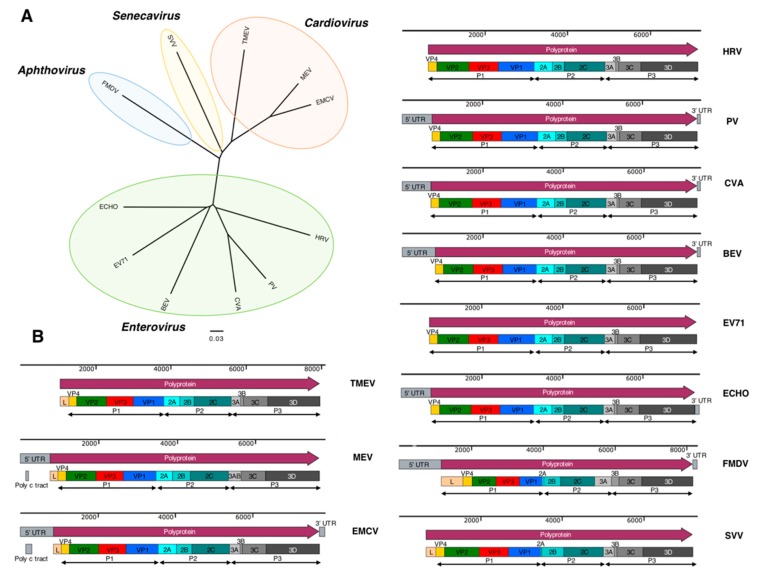
Genome-based phylogeny of different picornaviruses. (**A**) A radial phylogenetic tree demonstrating the evolutionary relationship among some members of *Enterovirus*, *Aphthovirus*, *Senecavirus* and *Cardiovirus* genera. Complete RNA sequences were aligned using Clustal Omega. Multiple alignment tool, scale bar representing number of nucleotide changes per site [29]. (**B**) RNA genome, polyprotein and cleaved mature peptides of different picornaviruses. Picornaviruses contain a plus-strand RNA genome, which is approximately ~7.1–8.9 kb in size. RNA genome codes for a polyprotein, which is organized into regions, P1, P2 and P3, with the leader protein present in some picornaviruses. P1 encodes for four structural proteins, VP1, VP2, VP3 and VP4. P2 and P3 encodes for 2A–C and 3A–D non-structural proteins, respectively. NCBI accession codes for viruses are as follows: Human Rhinovirus, HRV (K02121.1), Poliovirus, PV (AF111984.2), Coxsackievirus A, CVA (AF546702.1), Bovine Enterovirus, BEV (NC_001859.1), Enterovirus 71, EV71 (KJ686308.1), Echovirus, ECHO (AF029859.2), Foot-and-Mouth virus, FMDV (DQ989323.1), Seneca Valley virus, SVV (NC_011349.1), Theiler’s Murine Encephalomyelitis virus, TMEV (M20301.1), Mengovirus, MEV (L22089.1), Encephalomyocarditis, EMCV (X74312.1).

The P2 region of the translated polyprotein consists of 2A, 2B and 2C. The picornaviral 2A is a protein which can be absent, or in some cases present in more than one copy in the picornaviral genome. The 2A proteins can be organised into five groups based on function and conserved residues; these are: chymotrypsin-like protease, parechovirus-like, hepatitis-A-like, apthovirus-like and cardiovirus 2A proteins [26]. The roles of 2B, 2C and their precursor 2BC are not completely understood, but they were shown to be involved in the formation of virally induced vesicles [26]. The final segment of the picornaviral polyprotein is P3, comprising 3A, 3B, 3C and 3D. A function for 3A beyond acting in concert with 3B is not known, but the hydrophobic carboxy terminus is thought to anchor the protein to membranes [27]. The 3B, also known as VPg is a small protein which associates with the 5′ terminus of the genome and plays an essential role in genome replication by providing a primer for RNA synthesis when uridylated by cis-acting replicative elements (CREs). The CREs are looped secondary structural elements which can occur at several places in the RNA genome, including 5′ and 3′ UTRs, and 2C regions. The protease encoded by 3C performs most of the cleavages of the picornaviral polyprotein as well as inhibiting host transcription. Last among the picornaviral proteins is 3D, the RNA-dependent RNA polymerase (RdRp) [28]. The joint 3C and 3D proteins, known as the 3CD protein, have protease and CRE binding activity, but do not have RdRp activity. Finally, the 3′ UTR of picornaviruses will have a poly-A tail and, occasionally, as previously mentioned, a CRE [27]. The phylogenetic relationship and genome structure of the picornaviruses discussed in this review is presented in Figure 3 [29].

By virtue of their distinct biology, picornaviruses possess several advantages for their development for cancer therapy. The small size of the capsid, around 30 nm, are an advantage in penetrating the blood–brain barrier. Picornaviral RNA genomes replicate in the cytosol and do not integrate into host chromosomes, so they are not genotoxic. Additionally, they do not encode oncogenes, and their genomes are easily manipulated as cDNA [30]. This could be considered advantageous when compared to the DNA genomes and nuclear replication of viruses like Adenoviruses and Vaccinia virus. However, this disadvantage is compensated by DNA viruses by their capability to be easily modified in order to encode foreign proteins. Conversely, the inherently error-prone process of viral RNA replication, which is estimated at between 0.01 to 1 mutation per hypothetical 10,000 base genomes, means that picornaviral genomic stability could present a problem [31]. However, this instability could also be interpreted as being a safety feature that prevents the introduction of new viruses in the population. *Picornaviridae* is an extremely rich viral family, and the viruses considered for cancer therapy include both unaltered, naturally occurring viruses as well as viruses that have been extensively altered to either attenuate pathogenesis or increase oncolytic activity. Although picornaviruses represent a subset of oncolytic viruses, the picornaviruses discussed in this review have a diverse range of oncolytic activities and mechanisms that dictate their selectivity for cancer cells, as demonstrated in Table 2.

## 2. Coxsackievirus

The discovery of Coxsackievirus was due to attempts made in the 1940s to find a more economically feasible animal system than macaques for the study of Poliovirus from patient samples [32]. An atypical paralysis of unweaned “suckling” mice, due to skeletal muscle destruction rather than central nervous system (CNS) damage, indicated that the aetiological agent was not Poliovirus, with the filtrable agent being neutralised in human sera, and differentiated from other similar viruses by host range [32]. Today, Coxsackievirus species are divided into group A, comprising 23 serotypes which affect mainly skeletal muscle in murine models, and group B, which affects a broader range of tissues [33]. In humans, Coxsackieviruses cause mild upper respiratory tract infections. The primary cellular receptor employed by Coxsackievirus is intercellular adhesion molecule 1 (ICAM-1) [33]. An additional receptor, decay accelerating factor (DAF), is necessary but not sufficient for infection [33]. Alternatively, some members such as Coxsackievirus B3 (CVB3) use an alternative receptor, an immunoglobulin-like molecule called Coxsackievirus and Adenovirus receptor (CAR) [34]. Nine serotypes of Coxsackievirus have demonstrated oncolytic activity against an extensive range of cancers including but not limited to melanoma [34], multiple myeloma [35], breast [36], bladder [37] and prostate cancers [38].

Most of the studies investigating the use of Coxsackievirus in cancer therapy centre around Coxsackievirus A21 (CVA21), registered as CAVATAK [33]. The oncolytic potential of CVA21 has been interrogated in a breadth of cancers, including melanoma, multiple myeloma, breast cancer and bladder cancer.

Potential oncolysis of a panel of six melanoma cell lines was inferred by increased levels of ICAM-1 and DAF [34]. This was confirmed by CVA21 infecting and causing lysis in each of these cell lines at a multiplicity of infection (MOI) of 1. Furthermore, CVA21 did not infect MRC5 and RD cells, which do not express ICAM-1 and express low levels of DAF. On the other hand, due to its use of CAR as a cellular receptor, CVB3 could kill RD cells but not the melanoma or MRC5 cells. However, high ICAM-1 expression is not the only determinant of productive oncolytic infection, as primary cultures of ex-vivo melanoma tumours were shown to be highly susceptible to CVA21 infection, while peripheral blood lymphocytes (PBLs) permitted only background replication, despite similar ICAM-1 expression [34]. This is mirrored in another study in which two melanoma cell lines, SK-Mel-28 and ME4405, were infected with CVA21. SK-Mel-28 was 10 to 100 times more susceptible than ME4405, although they express comparable levels of ICAM-1. CVA21 infection can be ablated with anti-ICAM-1 antibody blockade, and anti-DAF antibodies can block 50% of CVA21 infection of ME4405, with SK-Mel-28 being unaffected. Despite higher Sk-Mel-28 susceptibility to CVA21, Sk-Mel-28 and ME4405 share similar virus replication kinetics [39]. NOD-SCID mice with melanoma xenografts cleared tumours by day 21 post-infection when given 10^3^ TCID_50_ CVA21 (TCID—Tissue culture Infective Dose) and remained tumour-free until the end of the trial at day 35 [34]. CVA21 was able to exert significant antitumour effects in both SK-Mel-28 and ME4405 xenograft tumours regardless of whether injected intratumourally, intraperitoneally or intravenously [34,39]. Recent in vivo studies focused on CVA21 in combination with other therapies. One study, performed in C57BL mice bearing ICAM-1 expressing B16 melanoma tumours, showed that the therapeutic effect of eight intratumoural injections of CVA21 is increased when combined with four intraperitoneal deliveries of antimouse-PD-1 antibodies than either therapy alone [40]. Another trial used the same melanoma mouse model but with the treatment regime changed to four cycles of CVA21 and four cycles of anti-cytotoxic T-lymphocyte-associated antigen protein 4 (CTLA-4) antibodies [41]. This reinforced the results of the combination therapy exceeding the performance of either monotherapy, including on secondary challenge of tumour clearing mice with B16 cells [3].

High ICAM-1 and DAF expression was confirmed in three multiple myeloma (MM) cell lines, with lower expression on peripheral mononuclear blood cells (PMBCs). MM cell lines showed cytopathic effect (CPE) on infection with CVA21 at a MOI as low as 0.2, increasing viral tires 100 to 1000-fold after 24 h. By contrast, CVA21 infection of PMBCs showed only modest CPE at MOI of 20 [35]. When treated with CVA21, MM cells from ex vivo patient bone samples were selectively killed, though some off-target death of healthy cells suggested a need for a refinement of dosing. Bone progenitor cells incubated with CVA21 showed some nonsignificant decrease when compared to phosphate buffered saline (PBS) controls [35].

CVA21 was investigated for oncolytic activity against breast cancer [36]. Of nine breast cancer cell lines, seven had high expression of ICAM-1 and DAF and two had moderate expression. An MOI of 100 induced extensive lytic destruction in all of the breast cancer cell lines and none of the controls. Signs of CPE were observed in breast cancer cells at up to 1000-fold lower MOI than that required for CPE in healthy cells. Infection of the breast cancer cell lines resulted in an increase of CVA21 viral titre by 0.5–4 log. Breast cancer mammospheres were similarly susceptible to CVA21 as the monolayers [36]. In SCID mice, the growth of pre-established T47D breast cancer xenograft tumours was arrested by 5 × 10^7^ TCID_50_ CVA21 with a decrease in treatment tumour mass of 17 ± 6% compared to an increase in tumour mass of 2422 ± 932% in controls 26 days post-intervention [36]. CVA21 was shown to be active against MDA-MB-231 tumours forming spontaneous metastases, with a significant reduction in primary tumour size observed after two weeks, and tumour-free mice by day 42 post-treatment. CVA21-treated mice maintained a high serum viral titre for the duration of the trial [36].

To evaluate the effect of CVA21 on bladder cancers, a panel of ten bladder cancer cell lines were classified based on susceptibility [37]. Six cell lines were designated as susceptible; two were less susceptible, showing evidence of infection only by confocal microscopy; and the remaining two were refractile to CVA21 infection. The susceptibility of bladder cancers to CVA21 was directly proportional to ICAM-1 expression, with one exception. Treatment with mitomycin C (MC) can increase the levels of ICAM-1 in cells. When treated with MC up to 0.5 µg/mL, three of the most susceptible bladder cancer cell lines showed an increase in ICAM-1 expression with no associated cell death [37]. Treatment with 0.5 µg/mL MC followed by CVA21 infection was tested in the same three susceptible cell lines and one refractive cell line. However, treatment with 0.5 µg/mL MC followed by CVA21 infection in refractive cells did not make them more susceptible to virus infection [37]. Combination therapy of MC and CVA21 increased viral replication in ex vivo bladder cancer samples and induced increased release of apoptotic markers in bladder cancer cell lines. CVA21 destruction of susceptible bladder cancer cell lines induces immunogenic cell death, as indicated by the markers endo-calreticulin and HMGB1 (high mobility group box-1) protein [37]. This effect was further corroborated in vivo. In a vaccination assay, C56BL/6 mice pretreated with CVA21-lysed MB49-ICAM-1 bladder cancer cells were effectively protected from later challenge with the same cells injected into the opposite flank, compared to 50% of control mice challenged with uninfected lysate [37]. This protective effect was found to be largely due to CD4+ T-cells, as shown by a follow up trial with an anti-CD4 antibody blockade which resulted in 82% of surviving mice succumbing to disease. Anti-CD8 blockade and NK (natural killer) cell depletion showed no effect [37].

The combination of CVA21 with four common chemotherapy agents was assessed on a panel including breast cancer, colorectal cancer, pancreatic cancer and healthy cell lines [41]. These examinations revealed that doxorubicin hydrochloride (DH) had a synergistic effect when combined with CVA21 against the cancer cell lines and only slightly increased cell death in healthy cells, with the exception of low MOI CVA21 treatment of healthy cells, where the effect was reversed [41]. Increased levels of active caspases in treated cancer cell lines suggested cell death was caused by apoptosis. Trials in SCID mice with pre-established MDA-MB-231 breast cancer tumours supported the conclusion that CVA21 works in synergy with DH, as the combination therapy extended the lives of mice and reduced tumours masses to a greater degree than either monotherapy [41].

In addition to native virus, the RNA of CVA21 transcribed from plasmid DNA has been shown to be cytotoxic on cell lines including hepatocellular carcinoma, lung cancer, rhabdomyosarcoma, and melanoma [42]. This was confirmed in vivo where SCID mice with KAS6/1 multiple myeloma xenografts were treated with CVA21 RNA, with CVA21 virions and RNase treated CVA21 RNA as controls. The CVA21 virus and RNA treatment groups both cleared tumours, with the virus group achieving this one to three days faster than the RNA group. Adverse effects were seen in both treatment groups, with all mice developing myositis. Modulation of the dose only affected antitumoural efficacy, and not myositis [42].

The oncolytic activity of CVA21 in a diverse range of cancers both in vitro and in vivo prompted several clinical trials [43]. A Phase I clinical trial of CAVATAK in Stage IV melanomas showed that two doses of 10^7^, 10^8^ or 10^9^ TCID_50_ CAVATAK were safe, and five out of nine patients showed transient or stable tumour mass reduction, or stabilisation of tumours. In a Phase II clinical trial of CAVATAK in late stage melanoma (CALM), 57 patients were treated with up to 3 × 10^8^ TCID_50_ CAVATAK intratumourally on days 1, 3, 5, 8 and 22, followed by injections once every three weeks for 18 weeks. This treatment regime resulted in no NCI CTCAE (National Cancer Institute common terminology criteria for adverse events) grade 3 or 4 events, with 38.6% of patients showing immune-related progression-free survival at six months, and 19.3% of patients showing durable objective responses [44]. In an extension to this study, the role of patient immunity was further explored. The number of tumour-infiltrating immune cells, especially CD8+ and PD-L1+ cells, increased in five of six patients tested. In addition, CVA21 injection into lesions where single- or double-line immune checkpoint blockades had stopped working was able to reconstitute immune cell infiltrates in all four patients tested [45].

In a Phase I/II clinical trial on systemic treatment of resistant malignancies (STORM), the enrolled patients with late-stage cancer were grouped into three CAVATAK dose cohorts, low (n = 3, 10^8^ TCID_50_), medium (n = 3, 3 × 10^8^ TCID_50_) and high (n = ~80, 10^9^ TCID_50_) [46,47]. The most recent published update of the trial is evaluating the safety of CAVATAK in combination with prembrolizumab. While the trial is still ongoing, preliminary results are encouraging, with only one grade 3 event and no dose-limiting toxicities [48].

Revisiting the study of CVA21 with mitomycin C (MC) against bladder cancer cell lines, a Phase I/II clinical trial of CAVATAK in non-invasive bladder cancers (CANON study) was undertaken [49]. The first stage of the study established the safety of CAVATAK at doses of either 10^8^ TCID_50_, 3 × 10^8^ TCID_50_, or one of each. The second part of the study tested the combination therapy of two doses of 3 × 10^8^ TCID_50_ CAVATAK with 10 mg MC. The study established the safety of CAVATAK and MC and recorded viral replication within tumours, complete tumour response and induced inflammation and apoptotic cell death [49].

CVA21/CAVATAK as an oncolytic agent has garnered the greatest amount of research among the coxsackieviruses. However, the infection of CVA21 in humans is common, and pre-existing adaptive immunity to this strain could result in premature clearing of the virus before being able to exert its therapeutic effect on tumours. This prompted an evaluation of alternatives to CVA21 with less prevalent rates of immunity.

Coxsackievirus serotypes A13, A15 and A18 (CVA13, CVA15 and CVA18) have all been shown to infect and productively replicate in multiple melanoma cell lines, while not infecting peripheral blood mononuclear cells (PMBCs) [50]. A study of mice bearing SK-Mel-28 melanoma tumours treated with the various Coxsackievirus serotypes revealed their differences in antitumour activity [48]. By 48 h post-infection, all five CVA18-infected mice and two out of five CVA15-infected mice had cleared tumours. No pre-existing immunity against any of the strains was detected in blood samples of melanoma patients, healthy controls or commercially pooled Immunoglobulin G (IgG), showing their possible utility in patients with pre-existing CVA21 immunity [50].

Of 28 enteroviral strains tested against 12 human cancer cell lines and a bone marrow stroma control cell line, Coxsackievirus strains B2 Ohio-1 (CVB2 Ohio-1), B3 Nancy (CVB3 Nancy) and B4 JBV (CVB4 JBV) stood out as causing cell death in A549 and LK-87 lung cancer cells with no toxicity in controls [51]. In a second screen against nine non-small cell lung cancer cell lines (NSCLC), CVB3 Nancy induced cytolysis in all. Infectivity against these cells was directly proportional to the sum of CAR and DAF expression and could be abrogated by siRNA knockdown of CAR. CVB3 Nancy infection causes immunogenic cell death by active secretion of ATP, expression of calreticulin and post-apoptotic HMGB1 protein release [51]. In nude mice, one dose could suppress A549 tumours, while five doses could clear tumours in 50% of mice. Similarly, five doses of CVB3 Nancy treatment cleared EBC-1 squamous cell carcinoma tumours and H1299 human adenocarcinoma tumours in all mice tested. CVB3 Nancy could spread to secondary tumours in mice bearing bilateral A549 tumours, suppressing growth in both compared to controls. Side-effects in treatment mice did include moderate hepatic dysfunction and mild myocarditis [51].

Four strains of Coxsackievirus B3 (Nancy, 31-1-93, H3 and PD) were tested against colorectal cell lines expressing varying levels of CAR and DAF [52]. Of the five cell lines tested, PD showed high/moderate effects in all, 31-1-93 showed high moderate effects in some and H3 and Nancy had very low infection rates, as measured by genome amplification and cell lysis. The basis for the greater oncolytic activity of the PD strain of CVB3 resides in the fact that it can use the *N*- and *6-O*-sulfated heparan sulfate (HS) as a cellular receptor [52]. HS is a polysaccharide highly expressed on colorectal cancer cells. While all the strains tested could suppress bilateral DLD1 colorectal adenocarcinoma xenograft tumours in mice, their toxicities also became apparent. Despite showing similar viral titres in treated tumours, mice treated with CVB 31-1-93 died by day six post-infection, and four out of six mice treated with CVB3 Nancy died by day eight [52]. CVB3 Nancy could be isolated in high titres from the heart and low titres from brain, spleen and kidney of dead mice. All CVB3 PD-treated mice were alive by day ten, with five considered as heathy as PBS-treated controls. CVB3 PD did, however, show adaptation to healthy cells, where the tissues of one treated mouse contained CVB3 PD with several capsid mutations [52].

Three endometrial cancer (EC) cell lines, Ishikawa, HEC-1-A and HEC-1-B, were shown to express the cellular receptors for CVB3. When infected by CVB3 2035A at an MOI of 10, there was extensive lytic destruction of all three cell lines and high titres of viral progeny produced after 48 h [53]. Mouse studies showed single infusions of CVB3 2035A could suppress Ishikawa and HEC-1-B tumours significantly, but not HEC-1-A tumours [53]. Escalation to five doses of CVB3 2035A eliminated Ishikawa and HEC-1-B tumours and significantly inhibited HEC-1-A tumours. In mice bearing bilateral HEC-1-B tumours, five doses of CVB3 2035A administered to primary tumours could eliminate them and suppress secondary tumours. Secondary tumour suppression was likely caused by viral spread through blood circulation, as viremia increased through the duration of the study up to day six [53]. Another experiment with bilateral HEC-1-A tumours showed that a single CVB3 2035A dose was enough to suppress both tumours with no recorded deaths or significant body weight changes [53]. The therapeutic effect of CVB3 2035A extended to ex vivo patient samples, with a 10%–40% decreased viability of cancer cells and no toxicity to normal controls [53].

Finally, Coxsackievirus B6 (CVB6) in the form of three non-pathogenic live enterovirus vaccine strains (LEV8, LEV14 and LEV15) was tested against healthy and cancer cell lines. LEV15 stood out as having very little infectivity in untransformed cell lines and varying infectivity in the cancer cell lines tested [54]. LEV15 was serially passaged in the least susceptible cell lines, RD rhabdomyosarcoma, MCF7 breast cancer and A431 epidermal carcinoma cell lines, or in varying combinations thereof. This produced four strains which acquired the ability to infect and replicate in the cell lines through which they had been repeatedly passaged, without losing pre-existing infectivity to other cell lines. The four strains were sequenced and found to have mutations that conferred bio-adaptation [54]. Nude mice with xenograft tumours of C33A cervical cancer, DU-145 prostate cancer, RD and MCF-7 cell lines were treated with two of the bio-adapted LEV15 strains, along with parental LEV15 as a reference and PBS as a control. LEV15-RD-7/MCF7-9 (LEV15 passaged seven times through RD cells and nine times through MCF7 cells) showed oncolytic activity against each of the tumour types, whereas LEV-15 only showed oncolytic activity against C33A and DU-145 [54].

## 3. Poliovirus

Poliovirus is the causative agent of poliomyelitis, a disease infamous for causing life-long disability and paralysis throughout human history. Infection with Poliovirus (PV) is usually asymptomatic or manifested as transient flu-like symptoms and/or gastroenteritis [76]. An estimated 1% to 2% of PV infection cases result in the virus spreading to afferent nerves, where cellular destruction results in poliomyelitis [76]. Cellular susceptibility to PV is dependent on the expression of CD155 cellular receptor, also referred in the literature as Necl5 (Nectin-like Protein 5) or occasionally PVR (Poliovirus receptor) [55]. The receptor-mediated cytotoxicity of Poliovirus was confirmed by the successful infection by all PV strains of nonhuman cells humanised by lentiviral transduction of CD155 [77]. CD155 is often upregulated in metastases, having a role in cellular motility and invasiveness [78]. Therefore, development of Poliovirus as a cancer therapy has largely been centred around retaining the receptor mediated oncolytic activity of the wild-type virus, while attenuating neurovirulence in order to prevent poliomyelitis in patients. Polioviruses with varying degrees of alteration have been evaluated for oncolytic activity, ranging from live attenuated poliovirus [56], poliovirus replicons [79], poliovirus with re-arranged cis-acting replication elements (CREs) [57] and chimeric rhino-/polioviruses [80].

Live Attenuated Poliovirus A vaccine containing the Sabin 1 strain (LAPV) has been tested for oncolytic activity in six human bone and soft tissue cancer cell lines expressing moderate to high levels of CD155, as well as one murine osteosarcoma [56]. All but one of the human cell lines showed dose-dependent apoptosis in response to LAPV infection [56]. In mice bearing HT1080 sarcoma xenografts, three doses of 10^6^ TCID_50_ LAPV were sufficient to control tumour growth by 40% and induced cancer cells apoptosis, as shown by terminal deoxynucleotidyl transferase dUTP nick end labelling (TUNEL) assay [56].

One way to attenuate PV neurotoxicity is by using replication incompetent Poliovirus 1 (PV1) replicons. Replicons are constructed by deleting the P1 region that encodes the capsid proteins [79]. Once inside the targeted cell, this PV1 engineered RNA genome is capable of replicating and causing cell lysis. However, the replicons cannot form full encapsidated virions to spread to other cells. The generation of full virions is conditioned by the presence of a complementing *Vaccinia* virus vector that provides the P1 region [79]. PV1 replicons have shown broad spectrum cytotoxicity against central nervous system (CNS)/non-CNS tumours, and ex vivo primary patient tumours. They have also demonstrated the ability to significantly prolong survival of murine glioblastoma models by infecting primary tumours as well as distal metastases [79].

The PV mutant A_133_Gmono-cre illustrates another strategy to neuro-attenuate oncolytic PV [57]. Initially, mono-CRE PV was designed by moving the cis-acting replication element (CRE) from the viral 2C protein to a spacer region adjacent to the internal ribosome entry site (IRES) of the 5′ untranslated region (5′ UTR) [57]. The rationale for the relocation to the specific spacer site in the 5′ UTR was that base substitutions in this region attenuated the wild-type PV but were not genomically stable [57]. In vitro and in vivo adapted mono-CRE PV isolates accumulated mutations allowing increased replication in neuro-2a neuroblastoma cells. One mutation, A_133_G, was present in both cases, and so A_133_Gmono-CRE PV was isolated and used in further studies. Along with pre-exposure to mono-CRE PV, four intratumoural injections of A_133_Gmono-CRE PV into neuro-2a xenograft tumours were sufficient to clear tumours in 82% of mice, and also effectively vaccinate against re-challenge with neuro-2a cells [57]. CD8+ T-cells isolated from neuro-2a immune mice had potent antitumour activity when transferred to mice with pre-established neuro-2a tumours, and the non-infectious lysate from in vitro infected A_133_Gmono-CRE PV cultures also achieved this effect [81]. These results emphasise the role of the virus and the host immune system in successful oncolytic virotherapy.

By far the most promising prospect for the use of PV in a cancer therapeutic setting is the chimeric virus, PVSRIPO. PVSRIPO (initially referred to as “PV1 (RIPOS)”) is the Sabin strain of PV1 (PVS) with IRES replaced with that of human Rhinovirus 2 (HRV2) [80,82]. The consequence of this is an attenuation in neuronal cells [80,83].

The basis for neuronal attenuation of PVSRIPO is multifactorial. Wild-type polioviral IRES binds the eIF4G:4A:4B complex (eukaryotic initiation factor 4G:4A:4B), unwinding the RNA, exposing the start codon and binding eIF3, while the HRV2 IRES does not in neuron lineage cells [84]. PVSRIPO neuro-attenuation is also partly due to the presence of double-stranded RNA binding protein 76 (DRBP76). DRBP76 binds the HRV2 IRES in the cytoplasm of neuronal cells, preventing PVSRIPO genome replication. In neoplastic cells, DRBP76 is restricted to the nuclear compartment; therefore, it does not inhibit the chimeric virus replication which takes place in the cytoplasm [84]. PVSRIPO may have an advantage in replicating in cancer cells due to the broad phenomenon of deregulation of mitogenic signalling cascades favouring cap-independent translation. For example, the activation of MAPK-interacting kinase has a downstream effect of repressing serine–arginine-rich protein kinase (SRPK), which has an important role in cap-dependent translation by acting on ITAFs (IRES trans-acting factor) [84]. The specific mechanism by which SRPK acts on viral translation in unknown [85].

PVSRIPO has demonstrated potent oncolytic activity in a wide range of cancer cell lines derived from gliomas [58], breast cancers [59,86], glioblastoma multiforme [87], melanomas [60], astrocytomas [88,89] and prostate cancers [59].

Infection of ex vivo glioma primary cultures derived from stage II–IV patients with PVSRIPO showed drastic CPE by 6 h and complete CPE by 12 h, resulting in viral titres comparable to those produced by established glioma cell lines [58]. The ex vivo primary cultures showed CD155 expression at similar levels with the original tumours, as well as established glioma cell lines [58].

CD155 expression in human breast cancer cells is relatively low [86]. However, on a panel of five established breast cancer cell lines and six ex vivo breast cancer primary cultures, CD155 was expressed to a higher degree than normal control cells [86]. The MCF-7/HER2-18 cell line was selected as a model to evaluate the oncolytic activity of PVSRIPO in cerebral metastatic CD155+ breast cancers. MCF-7/HER2-18 cells were injected intrathecally, to model neoplastic meningitis, or intracranially. In the neoplastic meningitis model, PVSRIPO treatment increased median survival time by 130% compared to UV-inactivated controls, with no significant difference between mice treated with a low dose of 10^7^ plaque formation units (PFU) or a high dose of 10^9^ PFU of PVSRIPO. In the intracranial model, the distinction between the administered doses of PVSRIPO was more pronounced, with the low dose extending median survival times by 122% and the high dose increasing median survival times by 153% compared to controls [86].

PVSRIPO was also tested in a glioblastoma mouse model [87]. Athymic mice bearing U87MGΔEGFR glioblastoma multiforme tumours were challenged with either a low dose of (10^7^ PFU) or high dose (10^9^ PFU) PVSRIPO. While there was no significant dose response relationship between the two dose cohorts, median survival compared to controls increased by 187.5% and 200%, respectively, with one of ten mice in the low dose cohort, and three of ten mice in the high dose cohort surviving to the end of the trial [87]. PVSRIPO was shown to have cytotoxicity against U87MG astrocytoma cells in the development and optimisation of a colorimetric assay to measure PVSRIPO viral lysis of cell lines in vitro, but not in HEK-293 embryonic kidney cells [88]. The activity of PVSRIPO against astrocytoma cells was the central focus of another study [89]. Athymic mice with bilateral HTB-15 astrocytoma tumours treated with 10^8^ TCID_50_ PVSRIPO in both tumours showed an average of a 45% decrease in tumour mass by day 10, nearly clearing the xenografts by day 28. Tumours isolated from mice sacrificed at day 10 and 28 showed a temporal progression towards tumour death, with day 10 tumours displaying a dense core of intact cancer cells surrounded by a loose mantle of dispersed tumour cell foci and immune infiltrates. At day 28, the tumours were composed primarily of scar tissue, with minor foci of heterogenous composition [89]. PVSRIPO was only isolated at low levels from day 10 tumours. When pooled and purified, it maintained the characteristic thermosensitive phenotype with two single nucleotide polymorphisms at positions 97 of the 5′ UTR and 1824 in VP3 [89].

In melanoma cells, PVSRIPO effectively evades the innate immune response, where other related viruses such as Enchephalomyocarditis virus (EMCV) cannot, and the mechanism of this is independent of melanoma differentiation-associated protein 5 (MDA5) and the mitochondrial antiviral signalling protein (MAVS) [60].

In vitro studies with SUM149 breast cancer cells and DU145 prostate cancer cells showed PVSRIPO had potent activity against these cell lines and that their infection released pro-inflammatory cytokines [59]. By day seven, single intratumoural injection of PVSRIPO in mice bearing orthotopic SUM149 and subcutaneous DU145 xenotransplants could reduce tumour weight, with some mice clearing tumours in both groups. Interestingly, PVSRIPO infection led to a massive increase in the recruitment of infiltrating neutrophils [59]. This highlights the contribution of the immune modulating activity of PVSRIPO to overall antitumour efficacy.

PVSRIPO has been shown to infect human THP1 macrophages, but unlike many of the cancer cells studied, PVSRIPO infection of macrophages is sublethal and serves to induce expression of major histocompatibility complex class II and costimulatory molecules and further leads to (interferon-β) IFN-β, (interleukin-12) IL-12 and (tumor necrosis factor- α)TNF-α production [90]. The immune stimulating effect could also be induced by virus-free lysate from PVSRIPO-infected tumours which activated human dendritic cells (DCs) to stimulate tumour-antigen specific T-cells [91]. PVSRIPO activation of DCs comes in part by a sublethal infection producing low viral progeny and is exaggerated when exposed to tumour lysate, as measured by CD40, CD80, IFN-β and TNF-α expression [90]. PVSRIPO oncolysis releases a medley of cancer antigens, including MART-1 (melanoma-associated antigen recognised by T-cells-1), DAMPS (damage-associated molecular patterns, e.g., heat shock protein 60/70/90, high mobility group box-1 protein) and double-stranded RNA (dsRNA) [91]. In mice, PVSRIPO adapted to mouse astrocytoma cells (mRIPO) was used for the treatment of transgenic melanoma tumours expressing ovalbumin (OVA), which produced cytotoxic T-lymphocytes (CTLs) primed against OVA and native melanoma antigen tyrosinase related protein 2. Infusion with mRIPO delayed tumour growth, increased animal survival times and induced tumour invasion of neutrophils, followed by DCs and T-cells [91].

Following the recommendations of the World Health Organisation (WHO) for putative Poliovirus vaccines, before starting human trials, PVSRIPO had to be tested in primates. Ex vivo cultures of macaque and human kidney cells showed no species-specific infectivity for either PVS or PVSRIPO [92]. Macaques treated with 10^7^, 10^9^ or 5 × 10^9^ TCID_5_ PVSRIPO showed no side-effects, and all survived to the end of the trial [92]. The virus was contained to the brain of all macaques with only one case that showed low viral titre in the spinal cord and pons/medulla. PVSRIPO was not shed in the serum, saliva, urine or faeces of infected animals. Antibodies against PVSRIPO could be isolated from day 10 and increased in titre by day 56 [92]. These results gave an optimistic prediction of the safety of PVSRIPO in humans. Indeed, a Phase I clinical trial in recurrent glioblastoma showed the safety of PVSRIPO in humans, with none of the 13 patient cohorts developing adverse events exceeding NCI CTCAE (National Cancer Institute common terminology criteria for adverse events) grade 3, and only one showing grade 4 event from a catheter removal [93,94]. A Phase I dose-finding study in 61 patients with recurrent glioblastomas indicated an optimum treatment dose of PVSRIPO of 5 × 10^7^ TCID_50_ [95]. In addition, PVSRIPO had a protective effect on patient survival, with 21% survival at 36 months compared to 4% in historical controls [95].

## 4. Echoviruses

Enteric cytopathic human orphan (ECHO) viruses are part of the species Enterovirus B. Several echoviruses have been evaluated for selective tropism towards different cancers, including EV7, EV1, EV5, EV12, EV15, EV17, EV26 and EV29.

Most abundant information regarding clinical applications is available on Echovirus-7 (EV7), registered by the name Rigvir [96]. Rigvir was discovered when the viromes of patients receiving the Salk inactivated Poliovirus vaccine were being monitored [96]. Rigvir derives its name from Riga, the capital of Latvia [97]. It is believed that in Latvia, 75% of melanoma cases are treated with Rigvir [98]. Other Eastern European countries such as Georgia, Armenia and Uzbekistan have also approved Rigvir [96]. The United States, much of the European Union and Japan found a lack of experimental evidence to approve Rigvir in cancer treatment [99].

Rigvir has shown toxicity in vitro against a broad range of cell lines, including melanoma, pancreatic adenocarcinoma, muscle rhabdomyosarcoma, mesenchymal stem cells, gastric carcinoma, lung carcinoma and human normal dermal fibroblasts [63].

Clinical trials are referred to in the literature to have occurred between 1965 and 1991, but the results from these clinical trials are not readily available [96]. Therefore, clinical data relating to the efficacy of Rigvir are largely based on retrospective studies and patient case studies from recent years rather than documented clinical trials.

One such retrospective study showed a statistically significant increase in 3-year survival of melanoma patients when treated with Rigvir post-surgery than surgery alone or with other immunomodulators [97]. For best efficacy, Rigvir is injected intratumourally rather than intramuscularly, increasing 5-year survival between 29.9% and 19.5% [97]. Regional inoculation of Rigvir increases the total proportion of active (CD38+) and cytotoxic (CD8+) T-cells [100]. Another retrospective trial focused on melanoma patients Stage IB through to Stage IIC, with 52 patients enrolled for Rigvir therapy and 27 electing to go without [97]. The Rigvir treatment regime started with one injection per day, reaching one every 3 months by the end of the trial for a total of 32 injections. While no significant increase was observed in the time spent disease-free, the Rigvir group had lower mortality [97].

In recent times, case studies of Rigvir treatment in a diverse range of cancers have been emerging in the literature. One such study followed a stage IV melanoma, stage IIIA small-cell lung cancer and stage IV hystiocytic sarcoma on a long-term Rigvir treatment [98]. Following surgery, the stage IV melanoma patient was treated with Rigvir in combination with odansetron. The treatment consisted of initially frequent Rigvir injections that became less frequent as time progressed. The small-cell lung cancer patient was treated with lariphan and Rigvir but at a constant infrequent schedule for six years. In the case of the stage IV histiocytic sarcoma patient, Rigvir was administered as part of a therapeutic cocktail including multisite radiation therapy, doxorubicin, cyclophosphamide and helixor P. At the time of study publication, the condition of all three patients had improved and they were stable, with no clinical parameters exceeding National Cancer Institute Common Terminology Criteria for Adverse Events (NCI CTCAE) grade 1 [98].

Rigvir as a monotherapy and in combination with other therapies has also been tested in patients for whom the prognosis is especially poor. One patient with a basal cell carcinoma, a diagnosis with a median expected survival of five months, was treated with Rigvir post-surgery [101]. The treatment regime began with daily injections for three days, graduating step-wise to thrice-weekly for multiple years. At the time of publication, the patient’s condition has been stable for 3.9 years, with scans showing minimal residual metastasis [101]. Another study documented Rigvir treatment of a stage IV poorly-differentiated rectal adenocarcinoma, with a 5-year survival rate of 5% [102]. The patient was first treated with 11 injections of Rigvir in combination with eight infusions with folinic acid-fluorouracil-oxiplatin (FOLFOX-4) and four infusions of bevacizumab. This first stage of therapy supressed cancer metastases by 50%, allowing surgical resection. Post-surgery, the patient was given four final infusions of FOLFOX-4. Computerised Topography (CT) scans six years following the initial diagnosis showed cystic bodies on the liver, spleen, and kidneys, as well as fibrotic changes in the lungs, consistent with a complete response to therapy [102].

Other Echovirus species have also been investigated for activity against cancer. Echovirus 1 (EV1) has been tested for activity against ovarian, prostate and gastric cancer cell lines. VLA-2, also known as integrin *α*_2_*β*_1_, has been identified as the cellular receptor for EV1 [62]. Consistent with this finding, EV1 was shown to cause cytolysis in a panel of eight ovarian cancer cell lines and ovarian cell spheroids with high expression of *α*_2_*β*_1_. This effect could be ablated by treatment with anti-*α*_2_*β*_1_ antibodies [103]. EV1 was shown to be able to spread from one tumour to another and exert protective effects in mice bearing xenografts of ovarian OVHS-1 in the upper and lower flanks. While the control cohort had to be sacrificed due to disease progression after 2 weeks, EV1-treated mice lived to the end of the trial with two of five completely clearing tumours and the rest recording tumours less than 3 mm in diameter [103].

While several prostate cancer cell lines as well as healthy prostate cell lines were also shown to express *α*_2_*β*_1_, EV1 selectively destroyed the cancer cell lines [38]. Moreover, EV1 was shown to be effective against prostate tumours of markedly different sizes in vivo. In severe combined immune-deficient (SCID) mice, EV1 treatment of relatively small PC-3 tumours of 30–40 mm^3^ diameter was sufficient to reduce them to an average of 1 mm^3^ [100]. Different doses between 10^3^ and 10^7^ TCID_50_ were used to test the efficacy of EV1 in large LNCaP tumours of 300 mm^3^. The low dose of EV1 had a transient but significant protective effect, while the mid- and high doses of virus showed lasting significant tumour suppression [38].

The minimum infectious dose of EV1 to produce CPE in four gastric cancer cell lines expressing *α*_2_*β*_1_ was investigated [104]. These were between 3.3 × 10^−6^ to 3 × 10^−2^ TCID_50_/cell in MKN-45, AGS and NCI-N87 and >1 TCID_50_/cell in Hs746T. While Hs746T infection in vitro produced only minimal CPE and viral replication after 72 h, the other three cell lines produced a 100-fold increase in EV1 viral titre after 12 h [104]. MKN-45 cells expressing luciferase (MKN-45-Luc) were injected intraperitoneally into SCID mice and allowed to develop tumours over five days. At seven days post-infection, treatment with EV1 resulted in a dose-dependent decrease in tumour volume as measured by change in flux with the low dose (10^3^ TCID_50_) responsible for a 47.38% reduction, the medium dose (10^5^ TCID_50_) for a 68.5% reduction and a 71.5% reduction in the high dose (10^7^ TCID_50_) cohort. For reference, mice treated with PBS had an average flux increase of 28.75%, and most were sacrificed due to disease progression. Interestingly, circulating virus was initially different across dose groups, but then equalised [104].

Echovirus 5 (EV5) represents an interesting study into the oncolytic activity of the wild-type virus genome and the naked RNA transcribed from a cDNA clone [105]. EV5 was shown to replicate and efficiently destroy cancer cells and spheroids of colorectal origin [105]. In vitro transcription of cDNA clones of RNA viruses with either T3, T7 or SP6 RNA polymerases leads to the addition of several nongenomic nucleotides that have been shown to decrease infectivity compared to virions [105]. A construct made with a self-cleaving hammerhead ribozyme sequence directly upstream of the authentic genome was capable of restoring the native virus genome. This construct was found to be 20 times more infectious when transfected into HT29 colorectal cancer cells compared to uncleaved transcripts [105].

Finally, the broader oncolytic ability of Echoviruses was tested in a study with viruses including EV12, EV15, EV17, EV26, and EV29 against a selection of six colorectal cell lines [106]. EV12 attached and caused CPE in five out of six cell lines, with productive replication in four cell lines. EV15 only productively replicated in two cell lines. EV17 could bind all cancer cell lines and significantly increased viral titre in five cell lines. EV26 produced significant progeny virus in five cell lines and caused complete CPE in four of them. EV29 was able to bind in low levels to all cell lines tested and produce high amounts of progeny virus in five of them. Of note was that one cell line, LoVo, was resistant to infection by any of the Echoviruses tested. EV12, EV17, EV26 and EV29 were investigated for the ability to destroy HT29 spheroids, and all but EV29 were able to do so between five and nine days [106].

## 5. Bovine Enteroviruses

Bovine Enteroviruses are endemic in cattle, being routinely isolated from cow faeces, and are not pathogenic to humans [107]. Bovine Enterovirus 1 (BEV1) and Bovine Enterovirus 2 (BEV2) are classified as Enterovirus E and Enterovirus F, respectively [108]. They were investigated in the 1970s to 1990s for treatment miscellaneous neoplasms, including soft tissue and blood cancers [64,109].

BEV1 was first investigated for antitumour activity in 1971, wherein 65% of cancer cell lines met the threshold for significant viral killing by BEV1, compared to 4% of healthy cell lines [64]. The percentage of cancer cells death varied between 18% in Ehrlich ascites and 98% in L-cells, and this selectivity is thought to be receptor-based [64]. Sialic acids were suggested to have a role in viral adsorption, as treatment with *Vibrio cholera* neuraminidase (VCN) showed a dose- and time-dependent inverse correlation between VCN exposure and viral titre following BEV 261 infection in a panel of susceptible cancer cells [110].

Interestingly, whereas other picornaviruses can bio-adapt to cell lines (see: Echovirus 7), this could not be achieved with BEV1 [64]. The same study showed that BEV1 was able to completely eradicate sarcoma-1 xenograft tumours in mice and delayed death from leukaemia 4946 tumours by 48 h, showcasing the broad range of BEV1 cytotoxicity in cancer cell lines [64].

In addition to murine studies, BEV1 was also tested in rabbits and dogs and shown to be well tolerated [111]. Mice bearing ascites sarcoma 180 had a strong antitumour response when treated with BEV1, with splenic hyperplasia being the only side-effect. One dog with a seminoma had a transient response on single injection of BEV1, but not with a secondary dose [111]. Finally, rabbits injected with F-647a immortalised rabbit T-cells that developed rabbit adult T-cell-like leukaemia showed increased survival when given BEV MZ-468, with treatment animals surviving up to four months until the end of the trial, comparative to control animals which died by day 11 [109].

## 6. Seneca Valley Virus

Seneca Valley Virus (SVV) was originally discovered as a contaminant of cell culture [112]. The selective oncolytic activity of SVV was implied to be receptor-mediated, as transfection of resistant cells with SVV genomic RNA resulted in productive infection [113]. A genome-wide screen of SVV against haploid cells infected with a lentiviral library of single guide RNAs identified anthrax toxin receptor 1 (ANTXR1), also known as tumour endothelial marker 8 (TEM8) as the cellular receptor for SVV. The results were validated in H446 small-cell lung cancer (SCLC) cells. Moreover, ANTXR1 knockout mutations protected SVV-susceptible cells in vitro and in mice models, conclusively showing that the cellular receptor for SVV is ANTXR1 [65]. This was further corroborated by the elucidation of the structural basis for the interaction of SVV and ANTXR1 by cryo-electron microscopy [114]. Seneca Valley Virus (SVV) in the form of wild-type strain SVV-001 has been evaluated for selective infection and lysis of cancers including retinoblastoma [67], medulloblastoma [66], glioma [68] and small-cell lung cancer, reaching as far as Phase II clinical trials [115].

The oncolytic potential of SVV was first highlighted in a 2007 study [113]. Screening of a broad panel of cell lines showed susceptibility to SVV in 13 out of 23 SCLC cell lines, two non-small cell lung cancer (NSCLC) cell lines, seven of eight neuroendocrine paediatric cancer cell lines and two of three adrenal cell carcinomas. Importantly, only a few foetal cell lines and none of the adult noncancer cells were infected by SVV [113]. SVV is not a human pathogen; therefore, resistance to SVV is not common in humans, with only 1 in 50 pooled blood samples containing weakly neutralising antibodies [113]. Toxicity studies in mice showed no difference between treatment and control mice. Athymic mice treated with an excess of 10^8^ viral particles per kilogram (vp/kg) SVV could clear pre-established H446 xenograft tumours and most mice treated with 10^7^ vp/kg cleared tumours, with two exceptions. Similarly, when athymic mice with Y79 retinoblastoma xenograft tumours were treated with 10^8^, 10^11^ or 10^14^ vp/kg SVV, six of eight, seven of seven and five of seven mice, respectively, cleared the tumours [113]. This trial was concluded a decade before the identification of the cellular receptor, and it set the paradigm that SVV has oncolytic activity against cancers with neuroendocrine features.

Concurrently, SVV was shown to have activity against Weri retinoblastoma cells in vitro [67]. Doses as low as 0.5 viral particles per cell (vp/cell) were sufficient to kill 50% of target cells [67]. These findings were consistent with in vivo trials of immuno-deficient mice challenged with intraocular Y79 xenografts, where 19 of 20 tumours were successfully treated by a single injection of 10^13^ vp/kg SVV [67].

To research SVV in the application against medulloblastoma (MB), mouse MB models were established using ex vivo primary cultures isolated from 10 paediatric patients [66]. Primary cultures of xenograft cells were challenged with SVV in concentrations ranging from 0.3 to 66 MOI for 72 h. Five of the ten tumours were permissive to SVV infection, even at low MOI, and the other five were completely resistant. As cancer stem cells (CSCs) are known to be resistant to traditional therapies, green fluorescent protein (GFP) labelled SVV (SVV-GFP) was tested in cells sorted for the expression of stem cell marker CD133. CD133+ and CD133− cells were not differentially infected by SVV-GFP, regardless of cell line permissivity to virus. However, SVV exposure was shown to prevent the formation of neurospheres in permissive MB cell lines [66]. Furthermore, two types of aggressive anaplastic MBs permissive to SVV were xenografted into Rag2 SCID mice [66]. Established small and medium tumours were challenged with a single intravenous injection of SVV at a concentration of 5 × 10^12^ vp/kg. SVV treatment increased the survival times in mice of both tumour sizes. Of note is that SVV could cross the blood–brain barrier (BBB) and did not infect any healthy mouse brain cells [66].

Small-cell lung cancer (SCLC) is an aggressive type of tumour. There are two SCLC groups: classic and variant, which make up for 70% and 30%, respectively [70]. The variant phenotype of SCLC is distinct from the classic based on variable cell size, prominent nucleoli, high expression of genes associated with neuronal differentiation and a more aggressive phenotype [70]. SVV-GFP was tested in three classic and three variant ex vivo SCLC primary cultures [70]. While classic primary cultures were completely refractory, variant cultures were highly sensitive to SVV infection with EC_50_ (effective viral concentrations to cause lysis in 50% of cells) values of 1.6 × 10^−3^ vp/cell, 3.1 × 10^−4^ vp/cell and 3.9 × 10^−3^ vp/cell, respectively. Gene expression profiles of permissive and refractory cell lines showed that higher expression of late neurogenic transcription factor *NEUROD1* and lower expression of the early neurogenic transcription factor *ASCL1* correlated with permissivity to SVV [70]. Furthermore, mouse models challenged with classical SCLC xenografts were completely resistant to SVV, while the variant transplants were significantly suppressed for the duration of the trial. Investigations into SVV treatment dose showed that mice bearing LX36 tumours had similar responses of tumour suppression when treated with 10^9^ to 10^11^ vp/kg SVV, and two out of six mice completely cleared tumours when treated with 10^14^ vp/kg SVV. Kinetics studies showed peak replication of SVV occurred at day three [70].

Since SVV could exert a therapeutic effect in medulloblastoma models, it raised the question of its applicability in other cancers for which the BBB presents a significant obstacle, such as gliomas. In a study of six ex vivo glioma primary cultures, four were found permissive to SVV infection at an MOI as low as 0.5, while the other two remained resistant up to an MOI of 25 [68]. Replication studies of SVV-GFP in glioma neurospheres showed that not all permissive cell lines behaved identically, with maximal fluorescence occurring at day one for one cell line, at day two for two of the cell lines, and at day three for the final permissive cell line [68]. Three permissive cell lines and two resistant cell lines were injected intracranially in mice and allowed to form large tumours. At 48 h post-injection, SVV positive areas were evident in permissive tumours and increased in size by day seven, confirming the ability for SVV to cross the BBB [68]. A follow-up experiment evaluated the effect of SVV treatment on the average survival time with regard to tumour size [68]. This showed an overall increase of survival times for treatment mice in tumour models of permissive cell lines, and one of the initially resistant cell lines. The mice with medium tumours had a greater increase in average survival time, suggesting a role of intratumoural vasculature for efficacy of SVV treatment [68].

SVV-001 (the originally isolated strain of SVV) is registered by Neotropix inc. as NTX-010 [69]. A preclinical trial tested NTX-010 against 23 cell lines both in vitro and a cohort of 711 mice [69]. In nine of the 23 cell lines, over 90% of cells died when compared to controls. Especially susceptible cell lines belonged to rhabdomyosarcoma, Ewing sarcoma or neuroblastoma panels. In mice, objective responses to a single injection of 3 × 10^12^ vp/kg NTX-010 were seen in neuroblastoma, rhabdomyosarcoma, rhabdoid tumour, Wilms tumour and glioblastoma cell lines [69]. Taken together, these studies offered a strong body of evidence justifying clinical trials with SVV.

First, a phase I clinical trial of 30 patients with SCLC was undertaken [116]. Patients treated with NTX-010 between 10^7^ to 10^11^ vp/kg showed no dose-limiting toxicities, with flu-like symptoms mainly manifesting in the lower dose cohorts. Neutralising antibodies were detected as early as two weeks into treatment. In terms of outcomes, one patient showed disease stabilisation, while another five had minor responses, which were not sufficient to meet RECIST criteria (response evaluation criteria in solid tumours) [117]. The patient with stable disease was alive three years post-trial, up until the time of publication, with PET scans revealing a 50% decrease in tumours [116]. Shortly after, a second phase I clinical trial was launched in a cohort of children with neuroblastoma, rhabdomyosarcoma or other rare tumours with neuroendocrine features [118]. In part A of the trial, 13 patients were injected with 10^9^, 10^10^ or 10^11^ vp/kg NTX-010, and in part B, patients were given oral (days 1 to 14) and intravenous (days 1 and 8) cyclophosphamide, in combination with two doses of 10^11^ vp/kg NTX-010. The study showed that NTX-010 was well tolerated with a single dose-limiting toxicity event recorded. While no objective response was observed, six patients did show disease stabilisation. A rapid neutralising antibody response to NTX-010 was also shown in this trial [118]. Finally, a phase II double-blind clinical trial in patients with extensive stage SCLC that had been stable or responding to four cycles of platinum-based chemotherapy was established [115]. A cohort of 58 patients were randomised into Arm A, treated with a single dose of 1 × 10^11^ vp/kg NTX-010, or Arm B, the saline control. Grade 4 adverse events were seen in three of the Arm A patients and none of the Arm B. Between the two cohorts, there was no difference in progression-free survival, and overall survival was slightly less in Arm A (83%) vs. Arm B (85%). The trial was also prematurely terminated to investigate the death of one patient, which was confirmed to be unrelated to NTX-010 treatment [115]. It is important to take into consideration that all these studies were conducted before the identification of ANTXRI as the cellular receptor for SVV [115]. Based on this information, future clinical trials might select patients that could potentially benefit from a targeted SVV cancer therapy.

## 7. Theiler’s Murine Encephalomyelitis Virus

Theiler’s murine encephalomyelitis virus (TMEV) is a natural murine pathogen that has been engineered to express tumour antigens to induce a targeted antitumour immune response and thus act as a cancer vaccine.

TMEV engineered to express chicken ovalbumin (TMEV–OVA) can generate host immune responses against OVA-expressing tumours [119,120]. The expression of OVA in the leader protein comes at a distinct virulence cost to TMEV [121,122]. TMEV–OVA infection in susceptible strains of mice is attenuated compared to wild-type [121]. Despite attenuation, TMEV–OVA has been shown to generate increased levels of OVA-specific cytotoxic T-lymphocytes (CTLs) in vivo, increasing survival time and delaying tumour outgrowth in mice with established OVA-expressing B16 melanoma [121]. TMEV–OVA infection effectively cleared OVA-expressing tumour cells, but this process of tumour editing had allowed the growth of escape mutant tumours, which lost the expression of the targeted epitope [121]. While tumours could lose OVA expression, two different TMEV–OVA constructs were confirmed to be genetically stable in mouse models up to 21 days. OVA is widely used as a model antigen for its high immunogenicity, but authentic tumour antigen is often not as immunogenic. To confirm that the immunisation process can work for weakly immunogenic tumour antigen, TMEV expressing p66 was infected into a mouse breast cancer model, and three out of ten treated mice cleared tumours [122]. In highly immunogenic GL261-quad mouse glioma models, infection with TMEV–OVA intracranially or intraperitoneally could significantly increase OVA-specific CD8+ T-cell populations compared to wild-type TMEV infection. This increase in CTLs coincided with a significant delay of tumour outgrowth and increase in survival time for mice given TMEV expressing OVA. The requirement for CD8+ response for therapeutic effect was shown in perforin knockout mice bearing GL261-quad gliomas, which did not benefit from TMEV–OVA vaccination [119].

During the host immune response generated to TMEV expressing foreign antigen, the adaptive immune response targets a combination of the foreign antigen and TMEV antigens. TMEV is known to harbour a highly immunogenic region in the VP2 structural protein (VP2_121–130_) [120]. Suppression of the VP2_121–130_ region has been attempted in order to direct a greater proportion of the cytotoxic immune response to expressed foreign antigen. Deletion of this immunogenic region is shown to increase the relative amount of CTLs primed against foreign antigen [120]. Vector silencing, the inoculation of TMEV viral peptides before TMEV–OVA infection to induce tolerance, was investigated with the same aim [120]. However, this strategy seemed to have no appreciable effect on the magnitude of immune response directed toward OVA, tumour progression or overall survival of mice with melanoma or glioma [123]. In fact, TMEV–OVA infection without vector silencing appeared to be more efficacious in controlling tumour progression in the melanoma model [123].

Wild-type DA strain of TMEV has been investigated in vivo against breast cancer and melanoma cell tumours, without a significant protective effect in either [71]. Wild-type DA encoding RNA sequences from GDVII, a more neurovirulent subtype of TMEV, were used to make chimeric DA strain/GDVII subtype fusions [71]. Of these chimeric viruses, GD7-KS1, for which the GDVII insert replaced the 3′ end of the 5′ UTR to the 3′ end of the 2C protein of TMEV DA genome, was over 40 times more productive than the DA strain infection of B16 melanoma cells in vitro [71]. In vivo, GD7-KS1 significantly delayed B16 tumour outgrowth and increased survival when compared to DA strain and vehicle controls. Consistent with the observations from TMEV–OVA experiments [119,120], the number of CD8+ T-cell infiltrates increased markedly on GD7-KS1 infection, while infiltrating CD4+ T-cell numbers remained unchanged [71].

## 8. Encephalomyocarditis Virus and Mengovirus

Encephalomyocarditis virus (EMCV) was discovered in 1945 when a captive male gibbon suddenly and inexplicably died of pulmonary oedema and myocarditis [124]. Mice treated with filtered fluid from this oedema developed paralysis and died from myocarditis [124]. Somewhat similarly, Mengovirus (MEV) was originally isolated from a Rhesus monkey that developed hind leg paralysis [124]. MEV’s host range is incredibly wide, including voles, squirrels, elephants, swine, wild boar, racoons, antelope, lions, birds and several species of nonhuman primate [124]. Both EMCV and MEV fall within the Cardiovirus A species and, with limited literature on their use as oncolytic viruses, it is convenient to discuss them together. Encephalomyocarditis virus (EMCV) and Mengovirus (MEV) have had relatively little research conducted on their oncolytic activity. While wild-type EMCV had shown selective activity in sarcomas [74] and renal carcinomas [73] in vivo, MEV is only selectively toxic to multiple myelomas in vivo when novel attenuation methods are employed [75].

As far back as 1965, EMCV, referred to as Columbia-SK virus, showed oncolytic activity against fructose sarcomas in mice and rats, with a more pronounced effect in rats [74]. The interest in EMCV and MV as cancer therapeutics was reignited a half-century later, when EMCV’s potent activity against the retinoblastoma cell line Y79 was shown in vitro as well as in mice, with Y79 tumour reduction averaging 88.9% in size and 96.6% in weight [125]. EMCV was subjected to preclinical evaluation for the treatment of clear-cell renal cell carcinoma (CCRCC) based on the premise that these cells, which are resistant to apoptosis, will produce maximal virus progeny [73]. Indeed, inhibition of NFκB signalling reduced CCRCC susceptibility to EMCV by promoting apoptosis [73]. Conversely, interventions that increased NFκB signalling and suppressed apoptotic signalling, such as null mutations of tumour suppressor protein von Hippel–Lindau (VHL), were shown to produce 500-fold greater EMCV viral titres than analogous wild-type CCRCC cell lines. Moreover, VHL-null cells reconstituted with VHL could extend CCRCC cell line survival up to 24 h when infected with EMCV [73].

VHL is a tumour suppressor protein with a role in tumour necrosis factor (TNF)-α mediated cell apoptosis [126] and oxygen-dependent degradation of hypoxia-inducible factor (HIF)-α, which helps move cells to an anaerobic phenotype and assists survival [73,126]. SCID mice challenged with luciferase-expressing CCRCC intratumourally injected with live or inactivated EMCV showed a 100-fold decrease in luciferase bioluminescence and increase of tumour necrosis in live EMCV treated tumours compared to controls [73]. Finally, in a wider context of showing how RNase L triggers autophagy of subcellular components in response to viral infection, EMCV proved to be able to infect HeLa M cells [127].

Similar to Poliovirus, the research into oncolytic Mengovirus (MEV) focused around novel attenuation methods [75]. A study of vMC_24_, a poly-C truncated attenuated MEV, demonstrated oncolytic activity in a miscellaneous panel of cancer cell lines that included several multiple myeloma cells of human and murine origin [75]. vMC_24_ caused significant CPE in the majority of cancer cell lines tested [75]. Of the resistant cell lines, murine multiple myeloma MPC-11 showed to be the most resistant to vMC_24_ infection. BALB/c mice bearing MPC-11 tumours were treated with either a single intravenous (IV) injection of 10^7^ TCID_50_ vMC_24_ or intratumoural (IT) injection of 10^6^ TCID_50_ vMC_24_. While IV injection showed only minimal tumour suppression, IT administration of vMC_24_ resulted in a decreased tumour volume within five days. However, three mice in the IV group and two in the IT group developed paralysis, with thee more dying overnight, suggestive of a heart failure [75]. Therefore, vMC_24_ showed potential as oncolytic agent but with lethal toxicity. This prompted a search for novel strategies to attenuate vMC_24_, such as the incorporation into the viral genome of sequences complementary to healthy tissue specific micro-RNAs [75,128]. Multiple vMC_24_ variants with complementary micro-RNA sequences (miRT viruses) were generated. The miRT viruses had combinations of miR125b (expressed in brain tissue), miR124 (expressed in neurons), miR133 and miR208a (enriched in cardiomyoctyes). Among these, a construct labelled vMC_24_-NC stood out as having very low viral titres in the brain, spine and heart of experimentally infected C57BL/6 mice. vMC_24_-NC had two miR124 sequences in the 5′ UTR, and one miR133b and one miR208a in the 3′ UTR. In vivo experiments with mice bearing MPC-11 tumours showed that four of ten mice treated with vMC_24_-NC cleared tumours and two delayed tumour growth, with no negative side-effects. The mice treated with vMC_24_ had a rapid tumour regression, but seven out of ten developed paralysis. Furthermore, vMC_24_-NC was also shown to eliminate tumours in a dose-dependent manner while remaining genetically stable in vivo [75].

## 9. Conclusions

This review discussed picornavirus species investigated for anticancer activity (Table 1). These viruses differ in their specific mode of oncolysis, the volume of research conducted on them as well as their future clinical prospects. CAVATAK and PVSRIPO are leading the charge in picornaviral oncotherapy by quickly progressing through clinical trials, with a number of clinical trials in active recruitment [19] As not all of the clinical trials have associated literature, Table 3 surmises the current and completed clinical trials for the picornaviruses discussed in this review. The approval of CAVATAK and PVSRIPO will depend on the outcome of these clinical trials. While SVV is currently not under clinical investigation, the identification of its cellular receptor provides a criterion by which patients might be evaluated for potential therapeutic benefit from NTX-010 in the near future, provided there exists sufficient interest to restart clinical trials [65]. For over a decade now, Rigvir has been utilised as a cancer treatment in Eastern Europe. However, despite recently documented case studies and retrospective studies, the adoption of Rigvir by other countries would be necessarily predicated on successful clinical trials. The case for Rigvir is also undercut by the unavailability of data from clinical trials which are said to have happened in the 1960s–1990s [96]. The rest of the viruses discussed in this review have yet to be tested in patients. TMEV raised interest with the positive results in recent mouse trials of genetically modified virus for the treatment of cancers with which it shares exogenously expressed antigens [71]. A clinical application in the near future for either BEV1 or BEV2 in oncolytic virotherapy seems highly unlikely, as the most recent investigation was published in 1991 [109]. However, it is not unheard of that academic interest in an oncolytic virus can be restarted after a decades-long hiatus, as shown with the 50-year gap in oncolytic EMCV research [73]. Finally, for both EMCV and MEV, recent studies have been undertaken, with the most recent EMCV study being explicit in identifying as preclinical research [73]. Taken as a whole, this review is evidence of picornaviruses emerging as dynamic and promising tools within the oncolytic virotherapy field.

## Figures and Tables

**Table 1:** Promising oncolytic viruses.

Species	Genus	Intellectual Property	Engineered Properties	Stage in Development	Examples of Highest Phase Clinical Trials	
***Herpes simplex* virus**	Herpesvirus	T-VEC®	Expressing Granulocyte Macrophage Colony- Stimulating Factor	US FDA Approved	**Phase III** T-VEC vs. combination therapy with Prembolizumab in melanoma NCT02263508 • Active Combination with Cisplatin and Radiotherapy for head and neck cancer NCT01161498 • Terminated	**Phase III** T-VEC vs. Granulocyte Macrophage Colony Stimulating Factor in melanoma NCT00769704 • Completed Melanoma NCT01368276 • Completed
**Adenovirus**	Mastadenovirus	Oncorine®	E1b 55k gene deletion	Approved (China)	**Phase III** Combination with Endostatin against Non-Small-Cell Lung Cancer NCT02579564 • Completed	
**Adenovirus**	Mastadenovirus	ONCOS-102®	Expressing Granulocyte Macrophage Colony- Stimulating Factor	Clinical trials (Phase I/II)	**Phase II** Combination with and Pemetrexed or Cisplatin in unresectable pleural mesothelemia NCT02879669 • Active **Phase I/II** Combination with DCVAC/Pc in metastatic prostate cancer NCT03514836 • Active	**Phase I/II** Combination with Durvalumab in advanced peritoneal malignancies NCT02963831 • Active **Phase I** Malignant solid tumours NCT01598129 • Completed
**Vaccinia virus**	Orthopoxvirus	PexaVec®/JX594	Expressing Granulocyte Macrophage Colony- Stimulating Factor and deletion of thymidine kinase	Clinical trials (Phase II/III)	**Phase III** Combination with Sorafenib vs. Sorafenib monotherapy in hepatocellular carcinoma NCT02562755 • Active **Phase II** Unresectable hepatocellular carcinoma NCT01171651 • Completed	**Phase II** Combination with Metronomic Cyclophosphamide in breast cancer or soft tissue sarcoma NCT02630368 • Active Malignant melanoma NCT00429312 • Completed
**Measles virus**	Morbillivirus	MV-NIS®	Encoding Human Thyroidal Sodium Iodide Importer	Clinical trials (Phase II)	**Phase II** Combination with Cyclophosphamide in refractory multiple myeloma NCT02192775 • Active MV-NIS vs. combination with Cyclophosphamide in treating with recurrent/refractory multiple myeloma NCT00450814 • Active	**Phase II** MV-NIS Vs. MV-NIS infected mesenchymal stem cells in recurrent ovarian cancer NCT02068794 • Active MV-NIS vs. chemotherapy in ovarian, fallopian or peritoneal cancer NCT02364713 • Active
**Reovirus**	Orthoreovirus	Reolysin®	N/A	Clinical trials (Phase II/III)	**Phase III** Combination with Paclitaxel and Carboplatin in platinum refractory head and neck cancers NCT01166542 • Completed **Phase II** Advanced and metastatic breast cancer NCT01656538 • Completed	**Phase II** Bone and soft tissue sarcomas metastatic to the lung NCT00503925 • Completed Combination with Docetaxel and Prednisone vs. Doxitaxel and Prednisone in Prostate Cancer NCT01619813 • Completed

As registered at https://clinicaltrials.gov [19]. Abbreviations: DCVAC/Pc (Dendritic cells pulsed with killed LNCaP prostate cancer cells), MV-NIS (Measles Virus-Sodium Iodide Symporter), PexaVec (Pexastimogene devacirepvec), T-VEC (Talimogene Laheraparepvec).

**Table 2:** Receptors and tissue tropisms of oncolytic picornaviruses.

Species	Genus	Receptor		Cellular Entry	Examples of Susceptible Cancers	
**Coxsackievirus**	**Enterovirus**	ICAM-1 & DAF (CVA21) CAR (CVB3)	[33] [53]	Receptor mediated endocytosis	Melanoma (CVA21) Multiple Myeloma (CVA21) Breast Cancer (CVA21) Bladder Cancer (CVA21) Endometrial Cancer (CVB3)	[34] [35] [36] [37] [53]
**Poliovirus**	Enterovirus	Necl5/CD155/PVR	[55]	Receptor mediated endocytosis	Bone/Soft Tissue (LAPV) Neuroblastoma (A_133_G mono-cre PV) Glioma (PVSRIPO) Breast Cancer (PVSRIPO) Melanoma (PVSRIPO)	[56] [57] [58] [59] [60]
**Echovirus**	Enterovirus	DAF (EV7) VLA-2	[61] [62]	Receptor mediated endocytosis	Melanoma Rhabdomyosarcoma Adenocarcinoma Lung carcinoma Basal cell carcinoma	[63] [63] [63] [63] [63]
**Bovine Enterovirus**	Enterovirus	HLA-DR (suggested)	[64]	Receptor mediated endocytosis	Leukaemia Soft tissue sarcoma	[64] [64]
**Seneca Valley Virus**	Senecavirus	ANTXR1/TEM8	[65]	Receptor mediated endocytosis	Medulloblastoma Retinoblastoma Glioma Glioblastoma Small Cell Lung Cancer	[66] [67] [68] [69] [70]
**Theiler’s Murine Encephalomyelitis Virus**	Cardiovirus	Sialic acid moeties	[71]	Receptor mediated endocytosis	Melanoma Breast Cancer	[71] [71]
**Encephalomyocarditis virus & Mengovirus**	Cardiovirus	Sialoglycoprotein	[72]	Receptor mediated endocytosis	Renal Carcinoma (EMCV) Sarcoma (EMCV) Multiple Myeloma (MEV)	[73] [74] [75]

Abbreviations: ANTXR1 (Anthrax Toxin Receptor 1), CAR (Coxsackievirus and Adenovirus Receptor), CD155 (Cluster of Differentiation 155), CVA21 (Coxsackievirus A 21), CVB3 (Coxsackievirus B 3), DAF (Decay Accelerating Factor), HLA-DR (Human Leukocyte Antigen—DR isotype), Necl5 (Nectin-like Protein 5), PVR (Poliovirus Receptor), TEM8 (Tumour Endothelial Marker 8), VLA-2 (Very Late Antigen 2).

**Table 3:** Summary of the clinical development of the picornavirus species discussed in this review.

Species	Genus	Intellectual Property	Stage in Development	Registered Clinical Trials *	
**Poliovirus**	Enterovirus	PVSRIPO	Clinical Trials (Phase I)	Triple Negative Breast Cancer NCT03564782 • Active Recurrent malignant glioma NCT02968178 • Active Unresectable melanoma NCT03712358 • Active	Recurrent malignant glioma in children NCT03043391 • Active Recurrent glioblastoma NCT01491893 • Active
**Coxsackievirus**	Enterovirus	CAVATAK	Clinical Trials (Phase I/II)	Melanoma—Combination with pembolizumab NCT02565992 • Active Non-Muscle Invasive Bladder Cancer—Combination with mitomycin C NCT02316171 • Completed Advanced melanoma—Combination with ipilimumab NCT02307149 • Active Head and neck cancer NCT00832559 • Terminated Late-stage melanoma NCT01227551 NCT01636882 • Completed	Uveal melanoma—Combination with ipilimumab NCT03408587 • Active Melanoma NCT00438009 • Completed Solid tumours NCT00636558 • Completed Non-Small Cell Lung Cancer & Bladder Cancer—Combination with pembolizumab NCT02043665 • Active Non-Small Cell Lung Cancer—Combination with pembolizumab NCT02824965 • Active
**Echovirus**	Enterovirus	Rigvir	Approved (Eastern Europe)	N/A	
**Bovine Enterovirus**	Enterovirus	N/A	*In vivo* testing	N/A	
**Seneca Valley Virus**	Senecavirus	NTX-010	Clinical Trials (Phase II)	Paediatric Cancers—Combination with cyclophosphamide NCT01048892 • Completed Solid tumours with neuroendocrine features NCT00314925 • Completed	Extensive Stage Small-Cell Lung Cancer NCT01017601 • Terminated
**Theiler’s Murine Encephalomyelitis virus**	Cardiovirus	N/A	*In vivo* testing	N/A	
**Encephalomyocarditis virus & Mengovirus**	Cardiovirus	N/A	*In vivo* testing	N/A	

* As registered on https://clinical trials.gov [19].

## Supplementary Materials

The following are available online at https://www.mdpi.com/2072-6694/11/5/685/s1, Movie: morphs between depth-cued atomic structures of oncolytic picornaviruses.

## References

[1] Bray F., Ferlay J., Soerjomataram I., Siegel R.L., Torre L.A., Jemal A. (2018). Global cancer statistics 2018: GLOBOCAN estimates of incidence and mortality worldwide for 36 cancers in 185 countries. CA Cancer J. Clin..

[2] Chahlavi A., Todo T., Martuza R.L., Rabkin S.D. (1999). Replication-Competent Herpes Simplex Virus Vector G207 and Cisplatin Combination Therapy for Head and Neck Squamous Cell Carcinoma. Neoplasia.

[3] Yuan M., Wong Y., Au G., Shafren D. (2015). Combination of intravenously delivered cavatak (coxsackievirus A21) and immune-checkpoint blockade significantly reduces tumor growth and tumor rechallenge. J. Immunother. Cancer.

[4] Pelner L., Fowler G.A., Nauts H.C. (1958). Effects of concurrent infections and their toxins on the course of leukemia. Acta Medica Scand. Suppl..

[5] Hoster H.A., Zanes R.P., Von Haam E. (1949). Studies in Hodgkin’s syndrome; the association of viral hepatitis and Hodgkin’s disease; a preliminary report. Cancer Res..

[6] Southam C.M., Moore A.E. (1952). Clinical studies of viruses as antineoplastic agents, with particular reference to egypt 101 virus. Cancer.

[7] Georgiades J., Zielinski T., Cicholska A., Jordan E. (1959). Research on the oncolytic effect of APC viruses in cancer of the cervix uteri; preliminary report. Inst. Med. Morskiej Gdansku.

[8] Asada T. (1974). Treatment of human cancer with mumps virus. Cancer.

[9] Greig S.L. (2016). Talimogene Laherparepvec: First Global Approval. Drugs.

[10] Xia Z.-J., Chang J.-H., Zhang L., Jiang W.-Q., Guan Z.-Z., Liu J.-W., Zhang Y., Hu X.-H., Wu G.-H., Wang H.-Q. (2004). [Phase III randomized clinical trial of intratumoral injection of E1B gene-deleted adenovirus (H101) combined with cisplatin-based chemotherapy in treating squamous cell cancer of head and neck or esophagus]. Ai zheng = Aizheng = Chin. J. Cancer.

[11] Garber K. (2006). China Approves World’s First Oncolytic Virus Therapy For Cancer Treatment. J. Natl. Cancer Inst..

[12] Morozova V.V., Babkin I.V., Baikov I.K., Netesov S., Chumakov P.M., Tikunova N.V. (2012). Oncolytic enteroviruses. Mol. Boil..

[13] Brown M.C., Dobrikova E.Y., Dobrikov M.I., Walton R.W., Gemberling S.L., Nair S.K., Desjardins A., Sampson J.H., Friedman H.S., Friedman A.H. (2014). Oncolytic Polio Virotherapy of Cancer. Cancer.

[14] Vacchelli E., Eggermont A., Sautes-Fridman C., Galon J., Zitvogel L., Kroemer G., Galluzzi L. (2013). Trial watch: Oncolytic viruses for cancer therapy. Oncoimmunology.

[15] Mahalingam D., Fountzilas C., Moseley J., Noronha N., Tran H., Chakrabarty R., Selvaggi G., Coffey M., Thompson B., Sarantopoulos J. (2017). A phase II study of REOLYSIN® (pelareorep) in combination with carboplatin and paclitaxel for patients with advanced malignant melanoma. Cancer Chemother. Pharmacol..

[16] Abou-Alfa G.K., Galle P.R., Chao Y., Brown K.T., Heo J., Borad M.J., Luca A., Pelusio A., Agathon D., Lusky M. (2016). PHOCUS: A phase 3 randomized, open-label study comparing the oncolytic immunotherapy Pexa-Vec followed by sorafenib (SOR) vs SOR in patients with advanced hepatocellular carcinoma (HCC) without prior systemic therapy. J. Clin. Oncol..

[17] Diaz R.M., Galivo F., Kottke T., Wongthida P., Qiao J., Thompson J., Valdes M., Barber G., Vile R.G. (2007). Oncolytic Immunovirotherapy for Melanoma Using Vesicular Stomatitis Virus. Cancer Res..

[18] Lichty B.D., Power A.T., Stojdl D.F., Bell J.C. (2004). Vesicular stomatitis virus: Re-inventing the bullet. Trends Mol. Med..

[19] ClinicalTrials.gov. https://clinicaltrials.gov/.

[20] Goddard T.D., Huang C.C., Meng E.C., Pettersen E.F., Couch G.S., Morris J.H., Ferrin T.E. (2018). UCSF ChimeraX: Meeting modern challenges in visualization and analysis. Protein Sci..

[21] Rossmann M.G., Arnold E., Erickson J.W., Frankenberger E.A., Griffith J.P., Hecht H.-J., Johnson J.E., Kamer G., Luo M., Mosser A.G. (1985). Structure of a human common cold virus and functional relationship to other picornaviruses. Nat. Cell Boil..

[22] Bedard K.M., Semler B.L. (2004). Regulation of picornavirus gene expression. Microbes Infect..

[23] Svitkin Y.V., Imataka H., Khaleghpour K., Kahvejian A., Liebig H.D., Sonenberg N. (2001). Poly(A)-binding protein interaction with elF4G stimulates picornavirus IRES-dependent translation. RNA.

[24] Bostina M. (2019). Monoclonal antibodies point to Achilles’ heel in picornavirus capsid. PLoS Boil..

[25] Tuthill T.J., Groppelli E., Hogle J.M., Rowlands D.J. (2010). Picornaviruses. Curr. Top. Microbiol. Immunol..

[26] Yang X., Cheng A., Wang M., Jia R., Sun K., Pan K., Yang Q., Wu Y., Zhu D., Chen S. (2017). Structures and Corresponding Functions of Five Types of Picornaviral 2A Proteins. Front. Microbiol..

[27] Porter A.G. (1993). Picornavirus nonstructural proteins: Emerging roles in virus replication and inhibition of host cell functions. J. Virol..

[28] Cameron C.E., Oh H.S., Moustafa I.M. (2010). Expanding knowledge of P3 proteins in the poliovirus lifecycle. Future Microbiol..

[29] Sievers F., Wilm A., Dineen D., Gibson T.J., Karplus K., Li W., Lopez R., McWilliam H., Remmert M., Söding J. (2011). Fast, scalable generation of high-quality protein multiple sequence alignments using Clustal Omega. Mol. Syst. Biol..

[30] Poirier J.T., Reddy P.S., Idamakanti N., Li S.S., Stump K.L., Burroughs K.D., Hallenbeck P.L., Rudin C.M. (2012). Characterization of a full-length infectious cDNA clone and a GFP reporter derivative of the oncolytic picornavirus SVV-001. J. Virol..

[31] Stern A., Bianco S., Yeh M.T., Wright C., Butcher K., Tang C., Nielsen R., Andino R. (2014). Costs and benefits of mutational robustness in RNA viruses. Cell Rep..

[32] Dalldorf G., Sickles G.M. (1948). An Unidentified, Filtrable Agent Isolated From the Feces of Children With Paralysis. Science.

[33] Bradley S., Jakes A.D., Harrington K., Pandha H., Melcher A., Errington-Mais F. (2014). Applications of coxsackievirus A21 in oncology. Oncolytic Virotherapy.

[34] Shafren D.R., Au G.G., Nguyen T., Newcombe N.G., Haley E.S., Beagley L., Johansson E.S., Hersey P., Barry R.D. (2004). Systemic Therapy of Malignant Human Melanoma Tumors by a Common Cold-Producing Enterovirus, Coxsackievirus A21. Clin. Cancer Res..

[35] Au G.G., Lincz L.F., Enno A., Shafren D.R. (2007). Oncolytic Coxsackievirus A21 as a novel therapy for multiple myeloma. Br. J. Haematol..

[36] Skelding K.A., Barry R.D., Shafren D.R. (2009). Systemic targeting of metastatic human breast tumor xenografts by Coxsackievirus A21. Breast Cancer Res. Treat..

[37] Annels N.E., Arif M., Simpson G.R., Denyer M., Möller-Levet C., Mansfield D., Butler R., Shafren D., Au G., Knowles M. (2018). Oncolytic Immunotherapy for Bladder Cancer Using Coxsackie A21 Virus. Mol. Ther. - Oncolytics.

[38] Berry L.J., Au G.G., Barry R.D., Shafren D.R. (2008). Potent Oncolytic activity of human enteroviruses against human prostate cancer. Prostate.

[39] Au G., Lindberg A., Barry R., Shafren D. (2005). Oncolysis of vascular malignant human melanoma tumors by Coxsackievirus A21. Int. J. Oncol..

[40] Shafren D., Quah M., Wong Y., Andtbacka R.H., Kaufman H.L., Au G.G. (2014). Combination of a novel oncolytic immunotherapeutic agent, CAVATAK (coxsackievirus A21) and immune-checkpoint blockade significantly reduces tumor growth and improves survival in an immune competent mouse melanoma model. J. Immunother. Cancer.

[41] Skelding K.A., Barry R.D., Shafren D.R. (2012). Enhanced oncolysis mediated by Coxsackievirus A21 in combination with doxorubicin hydrochloride. Investig. New Drugs.

[42] Hadac E.M., Kelly E.J., Russell S.J. (2011). Myeloma Xenograft Destruction by a Nonviral Vector Delivering Oncolytic Infectious Nucleic Acid. Mol. Ther..

[43] Shafren D., Smithers B.M., Formby M. (2011). A phase I, open-label, cohort study of two doses of coxsackievirus A21 given intratumorally in stage IV melanoma. J. Clin. Oncol..

[44] Andtbacka R.H., Curti B.D., Hallmeyer S., Feng Z., Paustian C., Bifulco C., Fox B., Grose M., Shafren D. (2015). Phase II calm extension study: Coxsackievirus A21 delivered intratumorally to patients with advanced melanoma induces immune-cell infiltration in the tumor microenvironment. J. Immunother. Cancer.

[45] Andtbacka R., Curti B., Hallmeyer S., Feng Z., Paustian C., Bifulco C., Fox B., Große M., Davies B., Karpathy R. (2015). 3336 Phase II CALM extension study: Enhanced immune-cell infiltration within the tumour micro-environment of patients with advanced melanoma following intralesional delivery of Coxsackievirus A21. Eur. J. Cancer.

[46] Pandha H., Harrington K., Ralph C., Melcher A., Grose M., Shafren D. (2015). Phase I/II storm study: Intravenous delivery of a novel oncolytic immunotherapy agent, Coxsackievirus A21, in advanced cancer patients. J. Immunother. Cancer.

[47] Harrington K., Ralph C., Melcher A., Kaufman D., Grose M., Karpathy R., Shafren D., Pandha H., Schmidt E. (2016). Intravenous coxsackievirus A21 in combination with pembrolizumab in advanced cancer patients: Phase Ib KEYNOTE 200 study. Ann. Oncol..

[48] Pandha H.S., Ralph C., Harrington K., Curti B.D., Sanborn R.E., Akerley W.L., Gupta S., Rudin C.M., Rosenberg J.E., Kaufman D.R. (2017). Keynote-200 phase 1b: A novel combination study of intravenously delivered coxsackievirus A21 and pembrolizumab in advanced cancer patients. J. Clin. Oncol..

[49] Annels N., Mostafid H., Sandhu S., Harrington K., Melcher A., Mansfield D., Au G., Karpathy R., Shafren D., Pandha H. (2016). Phase I/II CANON study: Oncolytic immunotherapy for the treatment of non-muscle invasive bladder (NMIBC) cancer using intravesical coxsackievirus A21. Ann. Oncol..

[50] Au G.G., Beagley L.G., Haley E.S., Barry R.D., Shafren D.R. (2011). Oncolysis of malignant human melanoma tumors by Coxsackieviruses A13, A15 and A18. Virol. J..

[51] Miyamoto S., Inoue H., Nakamura T., Yamada M., Sakamoto C., Urata Y., Okazaki T., Marumoto T., Takahashi A., Takayama K. (2012). Coxsackievirus B3 Is an Oncolytic Virus with Immunostimulatory Properties That Is Active against Lung Adenocarcinoma. Cancer Res..

[52] Hazini A., Pryshliak M., Brückner V., Klingel K., Sauter M., Pinkert S., Kurreck J., Fechner H. (2018). Heparan Sulfate Binding Coxsackievirus B3 Strain PD: A Novel Avirulent Oncolytic Agent Against Human Colorectal Carcinoma. Hum. Gene Ther..

[53] Lin Y., Wang W., Wan J., Yang Y., Fu W., Pan D., Cai L., Cheng T., Huang X., Wang Y. (2018). Oncolytic activity of a coxsackievirus B3 strain in human endometrial cancer cell lines. Virol. J..

[54] Svyatchenko V.A., Ternovoy V.A., Kiselev N.N., Demina A.V., Loktev V.B., Netesov S.V., Chumakov P.M. (2017). Bioselection of coxsackievirus B6 strain variants with altered tropism to human cancer cell lines. Arch. Virol..

[55] Strauss M., Filman D.J., Belnap D.M., Cheng N., Noel R.T., Hogle J.M. (2015). Nectin-Like Interactions between Poliovirus and Its Receptor Trigger Conformational Changes Associated with Cell Entry. J. Virol..

[56] Atsumi S., Matsumine A., Toyoda H., Niimi R., Iino T., Nakamura T., Matsubara T., Asanuma K., Komada Y., Uchida A. (2012). Oncolytic virotherapy for human bone and soft tissue sarcomas using live attenuated poliovirus. Int. J. Oncol..

[57] Toyoda H., Yin J., Mueller S., Wimmer E., Cello J. (2007). Oncolytic Treatment and Cure of Neuroblastoma by a Novel Attenuated Poliovirus in a Novel Poliovirus-Susceptible Animal Model. Cancer Res..

[58] Merrill M.K., Bernhardt G., Sampson J.H., Wikstrand C.J., Bigner D.D., Gromeier M. (2004). Poliovirus receptor CD155–targeted oncolysis of glioma1. Neuro-oncology.

[59] Holl E.K., Brown M.C., Boczkowski D., McNamara M.A., George D.J., Bigner D.D., Gromeier M., Nair S.K. (2016). Recombinant oncolytic poliovirus, PVSRIPO, has potent cytotoxic and innate inflammatory effects, mediating therapy in human breast and prostate cancer xenograft models. Oncotarget.

[60] Walton R.W., Brown M.C., Sacco M.T., Gromeier M. (2018). Engineered Oncolytic Poliovirus PVSRIPO Subverts MDA5-Dependent Innate Immune Responses in Cancer Cells. J. Virol..

[61] Plevka P., Hafenstein S., Harris K.G., Cifuente J.O., Zhang Y., Bowman V.D., Chipman P.R., Bator C.M., Lin F., Medof M.E. (2010). Interaction of Decay-Accelerating Factor with Echovirus 7. J. Virol..

[62] Bergelson J., Shepley M., Chan B., Hemler M., Finberg R. (1992). Identification of the integrin VLA-2 as a receptor for echovirus 1. Science.

[63] Tilgase A., Patetko L., Blāķe I., Ramata-Stunda A., Borodušķis M., Alberts P. (2018). Effect of the oncolytic ECHO-7 virus Rigvir® on the viability of cell lines of human origin in vitro. J. Cancer.

[64] Taylor M.W., Cordell B., Souhrada M., Prather S. (1971). Viruses as an Aid to Cancer Therapy: Regression of Solid and Ascites Tumors in Rodents After Treatment with Bovine Enterovirus. Proc. Natl. Acad. Sci. USA.

[65] Miles L.A., Burga L.N., Gardner E.E., Bostina M., Poirier J.T., Rudin C.M. (2017). Anthrax toxin receptor 1 is the cellular receptor for Seneca Valley virus. J. Clin. Investig..

[66] Yu L., Baxter P.A., Zhao X., Liu Z., Wadhwa L., Zhang Y., Su J.M., Tan X., Yang J., Adesina A. (2010). A single intravenous injection of oncolytic picornavirus SVV-001 eliminates medulloblastomas in primary tumor-based orthotopic xenograft mouse models. Neuro-oncology.

[67] Wadhwa L., Hurwitz M.Y., Chévez-Barrios P., Hurwitz R.L. (2007). Treatment of Invasive Retinoblastoma in a Murine Model Using an Oncolytic Picornavirus. Cancer Res..

[68] Liu Z., Zhao X., Mao H., Baxter P.A., Huang Y., Yu L., Wadhwa L., Su J.M., Adesina A., Perlaky L. (2013). Intravenous injection of oncolytic picornavirus SVV-001 prolongs animal survival in a panel of primary tumor–based orthotopic xenograft mouse models of pediatric glioma. Neuro-Oncology.

[69] Morton C.L., Houghton P.J., Kolb E.A., Gorlick R., Reynolds C.P., Kang M.H., Maris J.M., Keir S.T., Wu J., Smith M.A. (2010). Initial Testing of the Replication Competent Seneca Valley Virus (NTX-010) by the Pediatric Preclinical Testing Program. Pediatr. Blood Cancer.

[70] Dobromilskaya I., Poirier J.T., Moriarty W.F., Peacock C.D., Hann C.L., Rudin C.M. (2013). Selective tropism of Seneca Valley virus for variant subtype small cell lung cancer. J. Natl. Cancer Inst..

[71] Bell M.P., Pavelko K.D. (2016). Enhancing the Tumor Selectivity of a Picornavirus Virotherapy Promotes Tumor Regression and the Accumulation of Infiltrating CD8(+) T Cells. Mol. Cancer Ther..

[72] Jin Y.M., Pardoe I.U., Burness A.T., Michalak T.I. (1994). Identification and characterization of the cell surface 70-kilodalton sialoglycoprotein(s) as a candidate receptor for encephalomyocarditis virus on human nucleated cells. J. Virol..

[73] Roos F.C., Roberts A.M., Hwang I.I.L., Moriyama E.H., Evans A.J., Sybingco S., Watson I.R., Carneiro L.A.M., Gedye C., Girardin S.E. (2010). Oncolytic targeting of renal cell carcinoma via encephalomyocarditis virus. EMBO Mol. Med..

[74] Kuwata T. (1965). Infection of tumor cells by extraneous viruses. 3. Viral infection of tumors in homologous and heterologous hosts. Gan.

[75] Ruiz A.J., Hadac E.M., Nace R.A., Russell S.J. (2016). MicroRNA-Detargeted Mengovirus for Oncolytic Virotherapy. J. Virol..

[76] Mehndiratta M.M., Mehndiratta P., Pande R. (2014). Poliomyelitis: Historical facts, epidemiology, and current challenges in eradication. Neurohospitalist.

[77] Sosnovtseva A.O., Lipatova A.V., Grinenko N.F., Baklaushev V.P., Chumakov P.M., Chekhonin V.P. (2016). Sensitivity of C6 Glioma Cells Carrying the Human Poliovirus Receptor to Oncolytic Polioviruses. Exp. Boil. Med..

[78] Takai Y., Miyoshi J., Ikeda W., Ogita H. (2008). Nectins and nectin-like molecules: Roles in contact inhibition of cell movement and proliferation. Nat. Rev. Mol. Cell Boil..

[79] Ansardi D.C., Porter D.C., Jackson C.A., Gillespie G.Y., Morrow C.D. (2001). RNA replicons derived from poliovirus are directly oncolytic for human tumor cells of diverse origins. Cancer Res..

[80] Jahan N., Wimmer E., Mueller S. (2011). A Host-Specific, Temperature-Sensitive Translation Defect Determines the Attenuation Phenotype of a Human Rhinovirus/Poliovirus Chimera, PV1(RIPO). J. Virol..

[81] Toyoda H., Wimmer E., Cello J. (2011). Oncolytic poliovirus therapy and immunization with poliovirus-infected cell lysate induces potent antitumor immunity against neuroblastoma in vivo. Int. J. Oncol..

[82] Gromeier M., Alexander L., Wimmer E. (1996). Internal ribosomal entry site substitution eliminates neurovirulence in intergeneric poliovirus recombinants. Proc. Natl. Acad. Sci. USA.

[83] Cello J., Toyoda H., Dejesus N., Dobrikova E.Y., Gromeier M., Wimmer E. (2008). Growth phenotypes and biosafety profiles in poliovirus-receptor transgenic mice of recombinant oncolytic polio/human rhinoviruses. J. Med. Virol..

[84] Brown M.C., Gromeier M. (2015). Cytotoxic and immunogenic mechanisms of recombinant oncolytic poliovirus. Curr. Opin. Virol..

[85] Brown M.C., Bryant J.D., Dobrikova E.Y., Shveygert M., Bradrick S.S., Chandramohan V., Bigner D.D., Gromeier M., Lyles D.S. (2014). Induction of Viral, 7-Methyl-Guanosine Cap-Independent Translation and Oncolysis by Mitogen-Activated Protein Kinase-Interacting Kinase-Mediated Effects on the Serine/Arginine-Rich Protein Kinase. J. Virol..

[86] Ochiai H. (2004). Treatment of Intracerebral Neoplasia and Neoplastic Meningitis with Regional Delivery of Oncolytic Recombinant Poliovirus. Clin. Cancer Res..

[87] Ochiai H., Campbell S.A., Archer G.E., Chewning T.A., Dragunsky E., Ivanov A., Gromeier M., Sampson J.H. (2006). Targeted Therapy for Glioblastoma Multiforme Neoplastic Meningitis with Intrathecal Delivery of an Oncolytic Recombinant Poliovirus. Clin. Cancer Res..

[88] Yang X., Chen E., Jiang H., Muszynski K., Harris R.D., Giardina S.L., Gromeier M., Mitra G., Soman G. (2009). Evaluation of IRES-mediated, cell-type-specific cytotoxicity of poliovirus using a colorimetric cell proliferation assay. J. Virol. Methods.

[89] Dobrikova E.Y., Broadt T., Poiley-Nelson J., Yang X., Soman G., Giardina S., Harris R., Gromeier M. (2008). Recombinant Oncolytic Poliovirus Eliminates Glioma In Vivo Without Genetic Adaptation to a Pathogenic Phenotype. Mol. Ther..

[90] Brown M.C., Holl E., Boczkowski D., Walton R., Bigner D.D., Gromeier M., Nair S.K. (2015). Oncolytic poliovirus directs tumor antigen presentation and T cell activation in vitro. J. Immunother. Cancer.

[91] Brown M.C., Holl E.K., Boczkowski D., Dobrikova E., Mosaheb M., Chandramohan V., Bigner D.D., Gromeier M., Nair S.K. (2017). Cancer immunotherapy with recombinant poliovirus induces IFN-dominant activation of dendritic cells and tumor antigen-specific CTLs. Sci. Transl. Med..

[92] Dobrikova E.Y., Goetz C., Walters R.W., Lawson S.K., Peggins J.O., Muszynski K., Ruppel S., Poole K., Giardina S.L., Vela E.M. (2012). Attenuation of neurovirulence, biodistribution, and shedding of a poliovirus:rhinovirus chimera after intrathalamic inoculation in Macaca fascicularis. J. Virol..

[93] Desjardins A., Sampson J., Peters K., Vlahovic G., Threatt S., Herndon J., Boulton S., Lally-Goss D., McSherry F., Lipp E. (2014). Final Results of a Phase 1 Trial of an Oncolytic Polio/Rhinovirus Recombinant (Pvsripo) against Recurrent Glioblastoma (Gbm). Neuro-Oncology.

[94] Trotti A., Colevas A., Setser A., Rusch V., Jaques D., Budach V., Langer C., Murphy B., Cumberlin R., Coleman C. (2003). CTCAE v3.0: Development of a comprehensive grading system for the adverse effects of cancer treatment. Semin. Radiat. Oncol..

[95] Desjardins A., Gromeier M., Herndon J.E., Beaubier N., Bolognesi D.P., Friedman A.H., Friedman H.S., McSherry F., Muscat A.M., Nair S. (2018). Recurrent Glioblastoma Treated with Recombinant Poliovirus. N. Engl. J. Med..

[96] Alberts P., Tilgase A., Rasa A., Bandere K., Venskus D. (2018). The advent of oncolytic virotherapy in oncology: The Rigvir® story. Eur. J. Pharmacol..

[97] Donina S., Strele I., Proboka G., Auziņš J., Alberts P., Jonsson B., Venskus D., Muceniece A. (2015). Adapted ECHO-7 virus Rigvir immunotherapy (oncolytic virotherapy) prolongs survival in melanoma patients after surgical excision of the tumour in a retrospective study. Melanoma Res..

[98] Alberts P., Olmane E., Brokāne L., Krastiņa Z., Romanovska M., Kupčs K., Isajevs S., Proboka G., Erdmanis R., Nazarovs J. (2016). Long-term treatment with the oncolytic ECHO-7 virus Rigvir of a melanoma stage IV M1c patient, a small cell lung cancer stage IIIA patient, and a histiocytic sarcoma stage IV patient-three case reports. APMIS.

[99] Babiker H.M., Bin Riaz I., Husnain M., Borad M.J. (2017). Oncolytic virotherapy including Rigvir and standard therapies in malignant melanoma. Oncolytic Virotherapy.

[100] Bruvere R., Heisele O., Ferdats A., Rupais A., Muceniece A. (2002). Echovirus-mediated biotherapy for malignant tumours: 40 years of investigation. Acta Med. Litu.

[101] Proboka G., Tilgase A., Isajevs S., Rasa A., Alberts P. (2018). Melanoma Unknown Primary Brain Metastasis Treatment with ECHO-7 Oncolytic Virus Rigvir: A Case Report. Front. Oncol..

[102] Tilgase A., Olmane E., Nazarovs J., Brokāne L., Erdmanis R., Rasa A., Alberts P. (2018). Multimodality Treatment of a Colorectal Cancer Stage IV Patient with FOLFOX-4, Bevacizumab, Rigvir Oncolytic Virus, and Surgery. Case Rep. Gastroenterol..

[103] Shafren D.R., Sylvester D., Johansson E.S., Campbell I.G., Barry R.D. (2005). Oncolysis of human ovarian cancers by echovirus type 1. Int. J. Cancer.

[104] Haley E.S., Au G.G., Carlton B.R., Barry R.D., Shafren D.R. (2009). Regional administration of oncolytic Echovirus 1 as a novel therapy for the peritoneal dissemination of gastric cancer. J. Mol. Med..

[105] Israelsson S., Sävneby A., Ekström J.-O., Jonsson N., Edman K., Lindberg A.M. (2014). Improved replication efficiency of echovirus 5 after transfection of colon cancer cells using an authentic 5’ RNA genome end methodology. Investig. New Drugs.

[106] Israelsson S., Jonsson N., Gullberg M., Lindberg A.M. (2011). Cytolytic replication of echoviruses in colon cancer cell lines. Virol. J..

[107] Smyth M., Symonds A., Brazinova S., Martin J. (2002). Bovine enterovirus as an oncolytic virus: Foetal calf serum facilitates its infection of human cells. Int. J. Mol. Med..

[108] Adams M.J., King A.M.Q., Carstens E.B. (2013). Ratification vote on taxonomic proposals to the International Committee on Taxonomy of Viruses (2013). Arch. Virol..

[109] Shingu M., Chinami M., Taguchi T., Shingu M. (1991). Therapeutic Effects of Bovine Enterovirus Infection on Rabbits with Experimentally Induced Adult T Cell Leukaemia. J. Virol..

[110] Stoner G.D., Williams B., Kniazeff A., Shimkin M.B., Stoner B.W.G.D. (1973). Effect of Neuraminidase Pretreatment on the Susceptibility of Normal and Transformed Mammalian Cells to Bovine Enterovirus 261. Nat. Cell Biol..

[111] Hodes M.E., Morgan S., Hubbard J.D., Yu P.L., Lukemeyer J.W. (1973). Tissue culture and animal studies with an oncolytic bovine enterovirus (bovine enterovirus 1). Cancer Res..

[112] Hales L.M., Knowles N.J., Reddy P.S., Xu L., Hay C., Hallenbeck P.L. (2008). Complete genome sequence analysis of Seneca Valley virus-001, a novel oncolytic picornavirus. J. Virol..

[113] Reddy P.S., Burroughs K.D., Hales L.M., Ganesh S., Jones B.H., Idamakanti N., Hay C., Li S.S., Skele K.L., Vasko A.-J. (2007). Seneca Valley Virus, a Systemically Deliverable Oncolytic Picornavirus, and the Treatment of Neuroendocrine Cancers. J. Natl. Cancer. Inst..

[114] Jayawardena N., Burga L.N., Easingwood R.A., Takizawa Y., Wolf M., Bostina M. (2018). Structural basis for anthrax toxin receptor 1 recognition by Seneca Valley Virus. Proc. Natl. Acad. Sci. USA.

[115] Molina J. (2013). N-0923, a Randomized Double-Blind Phase Ii Study of the Seneca Valley Virus (Ntx-010) Vs Placebo for Patients with Extensive Stage Sclc (Es-Sclc) Who Were Stable or Responding after at Least 4 Cycles of Platinum-Based Chemotherapy: Alliance (Ncctg) Study. J. Thorac. Oncol..

[116] Rudin C.M., Poirier J.T., Senzer N.N., Stephenson J., Loesch D., Burroughs K.D., Reddy P.S., Hann C.L., Hallenbeck P.L. (2011). Phase I Clinical Study of Seneca Valley Virus (SVV-001), a Replication-Competent Picornavirus, in Advanced Solid Tumors with Neuroendocrine Features. Clin. Cancer Res..

[117] Eisenhauer E.A., Therasse P., Bogaerts J., Schwartz L.H., Sargent D., Ford R., Dancey J., Arbuck S., Gwyther S., Mooney M. (2009). New response evaluation criteria in solid tumours: Revised RECIST guideline (version 1.1). Eur. J. Cancer.

[118] Burke M.J., Ahern C., Weigel B.J., Poirier J.T., Rudin C.M., Chen Y., Cripe T.P., Bernhardt M.B., Blaney S.M. (2015). Phase I trial of Seneca Valley Virus (NTX-010) in children with relapsed/refractory solid tumors: A report of the Children’s Oncology Group. Pediatr. Blood Cancer.

[119] Renner D.N., Jin F., Litterman A.J., Balgeman A.J., Hanson L.M., Gamez J.D., Chae M., Carlson B.L., Sarkaria J.N., Parney I.F. (2015). Effective Treatment of Established GL261 Murine Gliomas through Picornavirus Vaccination-Enhanced Tumor Antigen-Specific CD8+ T Cell Responses. PLoS ONE.

[120] Bell M.P., Renner D.N., Johnson A.J., Pavelko K.D. (2014). A CD8 T-Cell Epitope Variant Enhances Immune Targeting to a Recombinant Picornavirus Vaccine Antigen. Viral Immunol..

[121] Pavelko K.D., Girtman M.A., Mitsunaga Y., Méndez-Fernández Y.V., Bell M.P., Hansen M.J., Allen K.S., Rodriguez M., Pease L.R. (2011). Theiler’s Murine Encephalomyelitis Virus as a Vaccine Candidate for Immunotherapy. PLoS ONE.

[122] Pavelko K.D., Bell M.P., Karyampudi L., Hansen M.J., Allen K.S., Knutson K.L., Pease L.R. (2013). The Epitope Integration Site for Vaccine Antigens Determines Virus Control While Maintaining Efficacy in an Engineered Cancer Vaccine. Mol. Ther..

[123] Malo C.S., Renner D.N., Kelcher A.M.H., Jin F., Hansen M.J., Pavelko K.D., Johnson A.J. (2016). The Effect of Vector Silencing during Picornavirus Vaccination against Experimental Melanoma and Glioma. PLoS ONE.

[124] Carocci M., Bakkali-Kassimi L. (2012). The encephalomyocarditis virus. Virulence.

[125] Adachi M., Brooks S.E., Stein M.R., Franklin B.E., Caccavo F.A. (2006). Destruction of human retinoblastoma after treatment by the E variant of encephalomyocarditis virus. J. Neuro-Oncology.

[126] Qi H., Ohh M. (2003). The von Hippel-Lindau tumor suppressor protein sensitizes renal cell carcinoma cells to tumor necrosis factor-induced cytotoxicity by suppressing the nuclear factor-kappaB-dependent antiapoptotic pathway. Cancer Res..

[127] Chakrabarti A., Ghosh P.K., Banerjee S., Gaughan C., Silverman R.H. (2012). RNase L Triggers Autophagy in Response to Viral Infections. J. Virol..

[128] Kelly E.J., Hadac E.M., Greiner S., Russell S.J. (2008). Engineering microRNA responsiveness to decrease virus pathogenicity. Nat. Med..

