# Correction: McCarthy, C.; et al. Developing Picornaviruses for Cancer Therapy. *Cancers* 2019, *11*, 685

**DOI:** 10.3390/cancers12030553

**Published:** 2020-02-28

**Authors:** Cormac McCarthy, Nadishka Jayawardena, Laura N. Burga, Mihnea Bostina

**Affiliations:** 1 Department of Microbiology and Immunology, University of Otago, Dunedin 9016, New Zealand; mccco791@student.otago.ac.nz (C.M.); jaygi915@student.otago.ac.nz (N.J.); 2 Otago Micro and Nano Imaging, University of Otago, Dunedin 9016, New Zealand

The authors wish to make the following corrections to this paper [1]:

On page 14, in paragraph two of Chapter 4, Echoviruses, the sentence: “The United States, much of the European Union and Japan found a lack of experimental evidence to approve Rigvir in cancer treatment [99]” should be deleted. 

On page 15, line one, the sentence: “One such retrospective study showed a statistically significant increase in 3-year survival of melanoma patients when treated with Rigvir post-surgery than surgery alone or with other immunomodulators [97]”, should be changed to: “One such retrospective study showed a statistically significant 4.39–6.57-fold decrease in mortality of melanoma patients when treated with Rigvir post-surgery than surgery alone [97]”. 

On page 15, line three, the sentence: “For best efficacy, Rigvir is injected intratumourally rather than intramuscularly, increasing 5-year survival between 29.9% and 19.5% [97]” should be deleted.

On page 15, paragraph two, line seven, the sentence “In the case of the stage IV histiocytic sarcoma patient, Rigvir was administered as part of a therapeutic cocktail including multisite radiation therapy, doxorubicin, cyclophosphamide and helixor P” should read: “In the case of the stage IV histiocytic sarcoma patient, Rigvir was administered for about 2 years followed by multisite radiation therapy, doxorubicin, cyclophosphamide and helixor P.”

On page 15, paragraph three, line two, the sentence “One patient with a basal cell carcinoma, a diagnosis with a median expected survival of five months, was treated with Rigvir post-surgery [101]” should read: “One patient with melanoma unknown primary brain metastasis, a diagnosis with a median expected survival of five months, was treated with Rigvir post-surgery [101].”

The authors would like to apologize for any inconvenience caused to the readers by these changes.
